# N-acetylcysteine modulates lipopolysaccharide-induced intestinal dysfunction

**DOI:** 10.1038/s41598-018-37296-x

**Published:** 2019-01-30

**Authors:** Sang In Lee, Kyung Soo Kang

**Affiliations:** 1 Department of Animal Resource and Science, Dankook University, Cheonan, Chungnam 330-714 Republic of Korea; 2 Bio Division, Medikinetics, Inc., Hansan-gil, Pyeongtaek-si, Gyeonggi-do, 17792 Republic of Korea

## Abstract

The gastrointestinal epithelium functions in nutrient absorption and pathogens barrier and its dysfunction directly affects livestock performance. N-Acetylcysteine (NAC) improves mucosal function, but its effects on intestinal functions at the molecular level remain unclear. Here, we performed gene expression profiling of the pig small intestine after dietary NAC treatment under LPS challenge and investigated the effects of NAC on intestinal epithelial cells *in vitro*. Dietary NAC supplementation under LPS challenge altered the small intestine expression of 959 genes related to immune response, inflammatory response, oxidation-reduction process, cytokine-cytokine receptor interaction, and the cytokine-mediated signalling, Toll-like receptor signalling pathway, Jak-STAT signalling pathway, and TNF signalling pathway. We then analysed the expression patterns of the top 10 altered genes, and found that NAC markedly stimulated *HMGCS3* and *LDHC* expression in IPEC-J2 cells. *In vitro*, NAC pre-treatment significantly reduced TNF-α and *NF-κB*, *TNF-α*, *IFN-γ*, and *IL-6* expression in LPS-induced IPEC-J2 cells. NAC treatment also significantly reduced oxidative stress in LPS-induced IPEC-J2 cells and alleviated intestinal barrier function and wound healing. Thus, NAC as a feed additive can enhance livestock intestinal health by modulating intestinal inflammation, permeability, and wound healing under LPS-induced dysfunction, improving our molecular understanding of the effects of NAC on the intestine.

## Introduction

The small intestinal epithelium is composed of a single layer of epithelial cells such as goblet cells and enterocytes that are tightly linked by tight and adherens junctions^1^. It plays a critical role in nutrient absorption via the transepithelial/transcellular pathway and in intestinal barrier function; it also serves as a barrier against pathogens and toxins, resulting in the regulation of the absorption of nutrients and macromolecules^2^. The functions of the intestinal epithelium are affected by various factors such as bacterial challenge and oxidative stress, resulting in intestinal dysfunction, which facilitates the transmission of pathogenic bacteria and toxins into the body^3^. Intestinal dysfunction is directly related to livestock growth performance such as feed intake, average daily gain, and gain/feed ratio in pigs^4^.

N-Acetylcysteine (NAC), a precursor of L-cysteine, has been widely used as an antioxidant, and it is rapidly metabolised by the small intestine to produce glutathione^5,6^. It is well accepted that NAC protects cells from oxidative damage by reacting with free radicals and interacting with ROS^7^. Thus, NAC indirectly exerts antioxidative effects via the synthesis of glutathione, which is essential for cellular defence against oxidative injury^7,8^. Furthermore, NAC improves mucosal function by reducing inflammation, alleviating oxidative stress, and ameliorating intestinal tissue damage by interacting with several intestinal cell signalling pathways, such as EGFR, TLR4, apoptosis, and tight junction signalling^9^.

Understanding the molecular mechanism underlying the modulation of intestinal function through several cell signalling pathways by NAC is a critical topic in livestock health. Thus, the present study performed gene expression profiling in the pig small intestine after dietary treatment with NAC under LPS challenge and evaluated the biological functions of NAC in IPEC-J2 cells.

## Results

### Identification of differentially expressed genes (DEGs)

**Figure 1:**
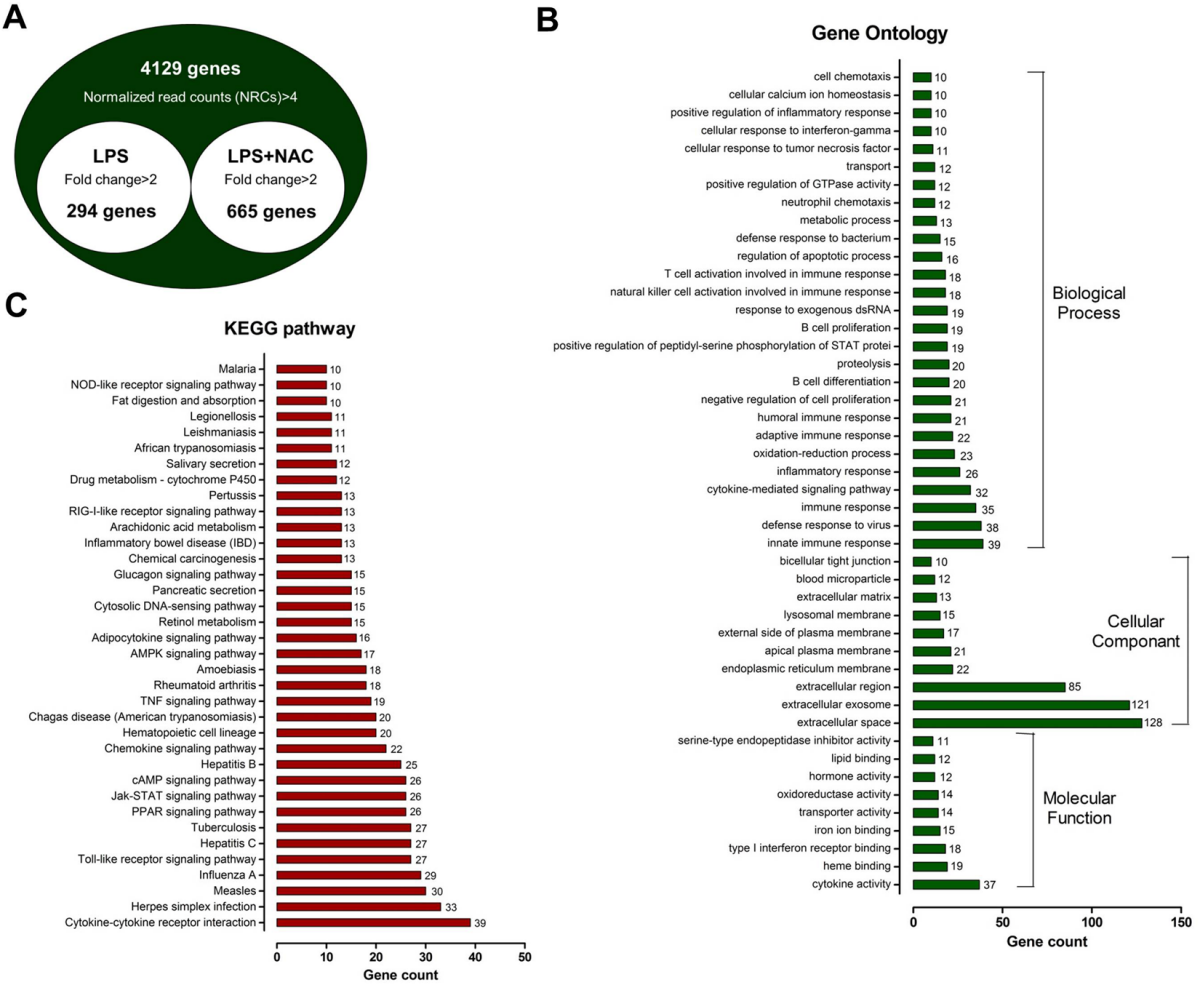
Gene expression profiling of the small intestine after dietary treatment with NAC for 21 d under LPS challenge. (**A**) Venn diagram illustrations of genes found to be differentially expressed with or without NAC treatment in the small intestine. The common genes from the RNAseq experiment were upregulated at least 2-fold (P < 0.05). (**B**) Gene Ontology (GO) enrichment analysis of differentially expressed genes. GO terms assigned to biological process, cellular component, and molecular functions. (**C**) Kyoto Encyclopaedia of Genes and Genomes (KEGG) pathway classification enrichment analysis of differentially expressed genes.

To identify DEGs affected by NAC treatment in pigs, we performed transcriptome analysis in the small intestine with or without NAC treatment for 21 d under LPS challenge; 959 DEGs were identified, among which 665 were upregulated and 294 were downregulated (Fig. 1A). These 959 DEGs were subjected to a gene ontology (GO) analysis comprising 27 biological processes, 10 cellular components, and 9 molecular functions (Fig. 1B and Supplemental Tables 1–3). The GO targets were associated with immune response, inflammatory response, cytokine-mediated signalling pathways, and oxidation-reduction processes. KEGG pathway analysis revealed that the DEGs were significantly enriched in the cytokine-cytokine receptor interaction, Toll-like receptor signalling pathway, Jak-STAT signalling pathway, and TNF signalling pathway (Fig. 1C and Supplemental Table 4). Moreover, we identified 692 annotated DEGs, among which 402 were upregulated and 292 were downregulated (Control vs LPS; Supplemental Fig. 1A). Six hundred and nine-two DEGs were subjected to a GO analysis comprising 20 biological processes, 8 cellular components, and 7 molecular functions (Supplemental Fig. 1B and Tables 5–7). DEGs related to the innate immune response, defence response to virus, immune response, cytokine-mediated signalling pathways, inflammatory response, oxidation-reduction processes, and negative regulation of cell proliferation were classified. KEGG pathway analysis revealed that the DEGs were significantly enriched in the cytokine-cytokine receptor interaction, neuroactive ligand-receptor interaction, and the PI3K-Akt signalling, Toll-like receptor signalling, Jak-STAT signalling, TNF signalling, RIG-I-like receptor signalling, TNF signalling, and NF-kappa B signalling pathways (Supplemental Fig. 1C and Table 8).

**Figure 2:**
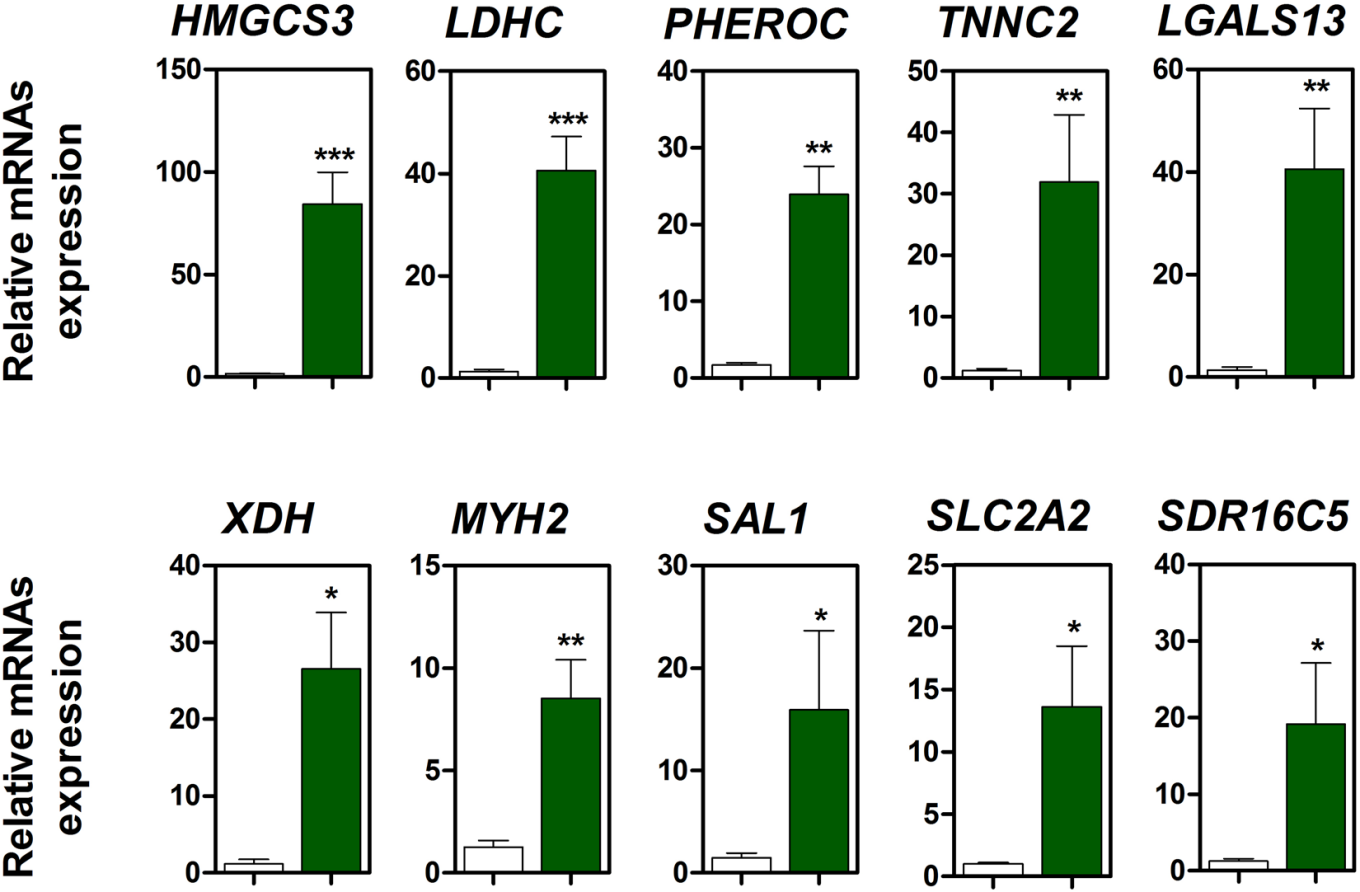
Quantitative expression analysis of genes that were highly expressed following NAC treatment in the small intestine (n = 3). Relative ratio between groups was normalized by *GAPDH* housekeeping gene. Error bars indicate standard errors (SEs) of triplicate analyses (***P < 0.001, **P < 0.01, and *P < 0.05).

To verify the expression of DEGs, we analysed the expression of the top 10 altered genes in NAC treated small intestine under LPS challenge (Fig. 2). *HMGCS3*, *LDHC*, *PHEROC*, *TNNC2*, *LGALS13*, *XDH*, *MYH2*, *SAL1*, *SLC2A2*, and *SDR16C5* (P < 0.05) were upregulated in the small intestine following NAC treatment compared with that without NAC treatment under LPS challenge (Fig. 2).

**Figure 3:**
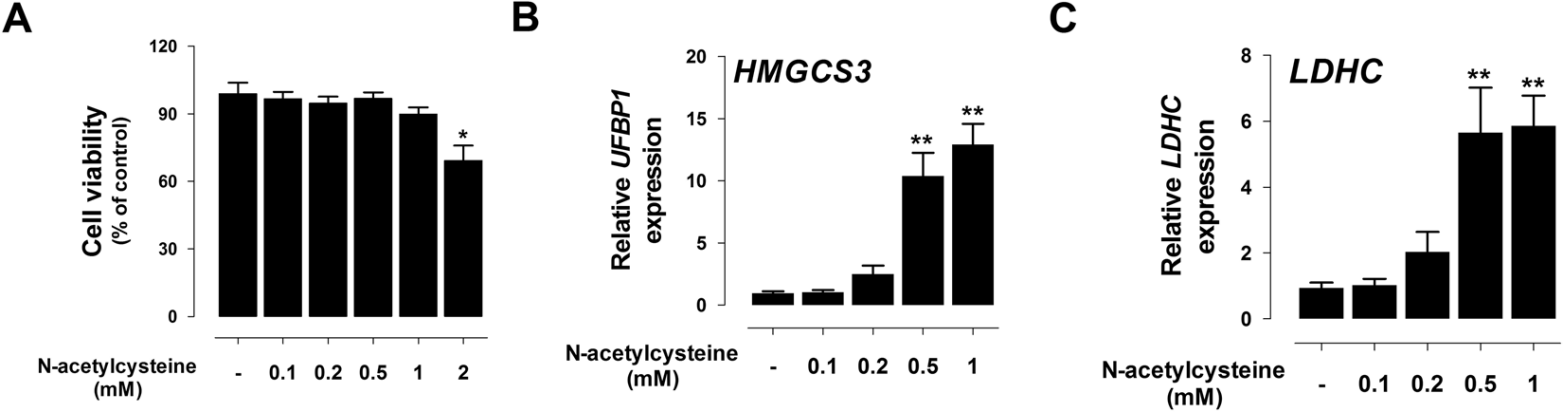
NAC regulated the expression of *HMGCS3* and *LDHC in vitro*. (**A**) Cytotoxicity against NAC treatment was determined. Various concentration of NAC (0 ~ 2 mM) were used to treat in IPEC-J2 cells for 24 h. Significant differences between the groups are described as ***P < 0.001, **P < 0.01, and *P < 0.05. Error bars indicate standard errors (SEs) of triplicate analyses. Expression of *HMGCS3* (**B**) and *LDHC* (**C**) was analysed with various concentration of NAC (n = 3).

To fine-tune the ideal dose of NAC in IPEC-J2 cells, we performed viability assay. Viability was decreased at treatment of 2 mM NAC for 48 h (Fig. 3A). In addition, the expression of *HMGCS3* and *LDHC* was investigated with various concentrations of NAC. After incubating IPEC-J2 cells for 48 h with various concentrations of NAC (0.1, 0.2, 0.5, and 1 mM), *HMGCS3* and *LDHC* mRNA levels were increased in a concentration-dependent manner. At concentrations of 0.5 and 1 mM, NAC markedly stimulated the expression of *HMGCS3* and *LDHC* in IPEC-J2 cells (P < 0.01; Fig. 3B,C). Based on these results, we used 0.5 mM NAC in subsequent experiments.

### Effect of NAC on inflammatory markers after LPS challenge in IPEC-J2 cells

**Figure 4:**
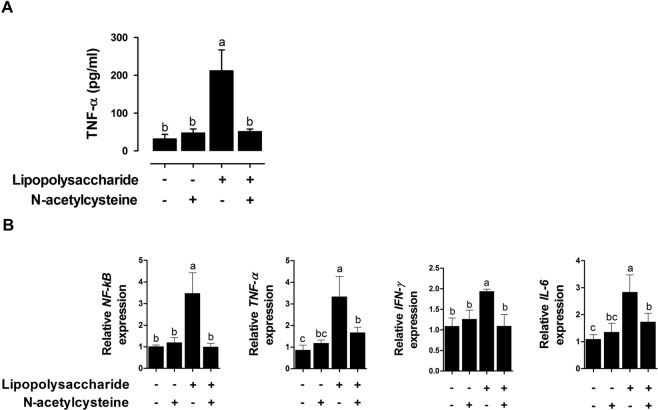
Effect of NAC on inflammatory markers after LPS challenge in IPEC-J2 cells (**A**) Effects of NAC treatment on the production of TNF-α after LPS challenge (n* = *3). (**B**) Quantitative expression analysis of genes encoding inflammatory cytokines after NAC treatment under LPS challenge (n = 3). Relative ratio between groups was calculated by the 2^−ΔΔCt^ method and normalized by *GAPDH* housekeeping gene. Error bars indicate standard errors (SEs) of triplicate analyses and lowercase letters represent significant differences (P < 0.05).

TNF-α production was evaluated to investigate immune reaction with or without NAC treatment under LPS challenge in IPEC-J2 cells. LPS challenge increased TNF-α production compared with that in the control (P < 0.05; Fig. 4A). Under LPS challenge, treatment with NAC significantly reduced TNF-α production (P < 0.05; Fig. 4A). However, treatment with NAC without LPS had no effect on TNF-α concentration in IPEC-J2 cells compared to that in the controls.

LPS challenge increased the expression of pro-inflammatory genes including *NF-κB*, *TNF-α*, *IFN-γ*, and *IL-6* compared with that in the control (P < 0.05; Fig. 4B). Under LPS challenge, treatment with NAC significantly reduced the expression of these genes (P < 0.05; Fig. 4B). However, treatment with NAC without LPS had no effect on the expression of pro-inflammatory genes in IPEC-J2 cells compared to expression in the control.

### NAC alleviates LPS-induced oxidative stress in IPEC-J2 cells

**Figure 5:**
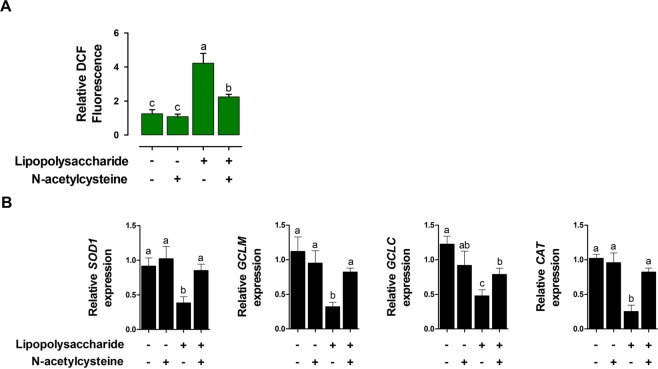
LPS-induced oxidative stress was alleviated by NAC treatment in IPEC-J2 cells. (**A**) NAC alleviated reactive oxygen species (ROS) generation in LPS-induced IPEC-J2 cells. ROS in the cells were detected using CM-H2DCFDA. (**B**) Quantitative expression analysis of genes that were related to oxidative stress after NAC treatment under LPS challenge (n = 3). Relative ratio between groups was calculated by the 2^−ΔΔCt^ method and normalized by *GAPDH* housekeeping gene. Error bars indicate standard errors (SEs) of triplicate analyses and lowercase letters represent significant differences (P < 0.05).

LPS challenge increased DCF fluorescence level as a marker of ROS production in IPEC-J2 cells compared with that in the control cells (P < 0.05; Fig. 5A). Under LPS challenge, treatment with NAC decreased DCF fluorescence (P < 0.05; Fig. 5A). However, treatment with only NAC had no effect on DCF level in IPEC-J2 cells compared with that in the control.

LPS challenge decreased the expression of oxidative stress-related genes including *SOD1*, *GCLM*, *GCLC*, and *CAT* compared with that in the control (P < 0.05; Fig. 4B). Under LPS challenge, treatment of NAC significantly increased the expression of these genes (P < 0.05; Fig. 4B). However, treatment with NAC without LPS had no effect on the expression of oxidative stress-related genes in IPEC-J2 cells compared with that in the control.

### NAC affects intestinal barrier function in LPS-treated IPEC-J2 cells

**Figure 6:**
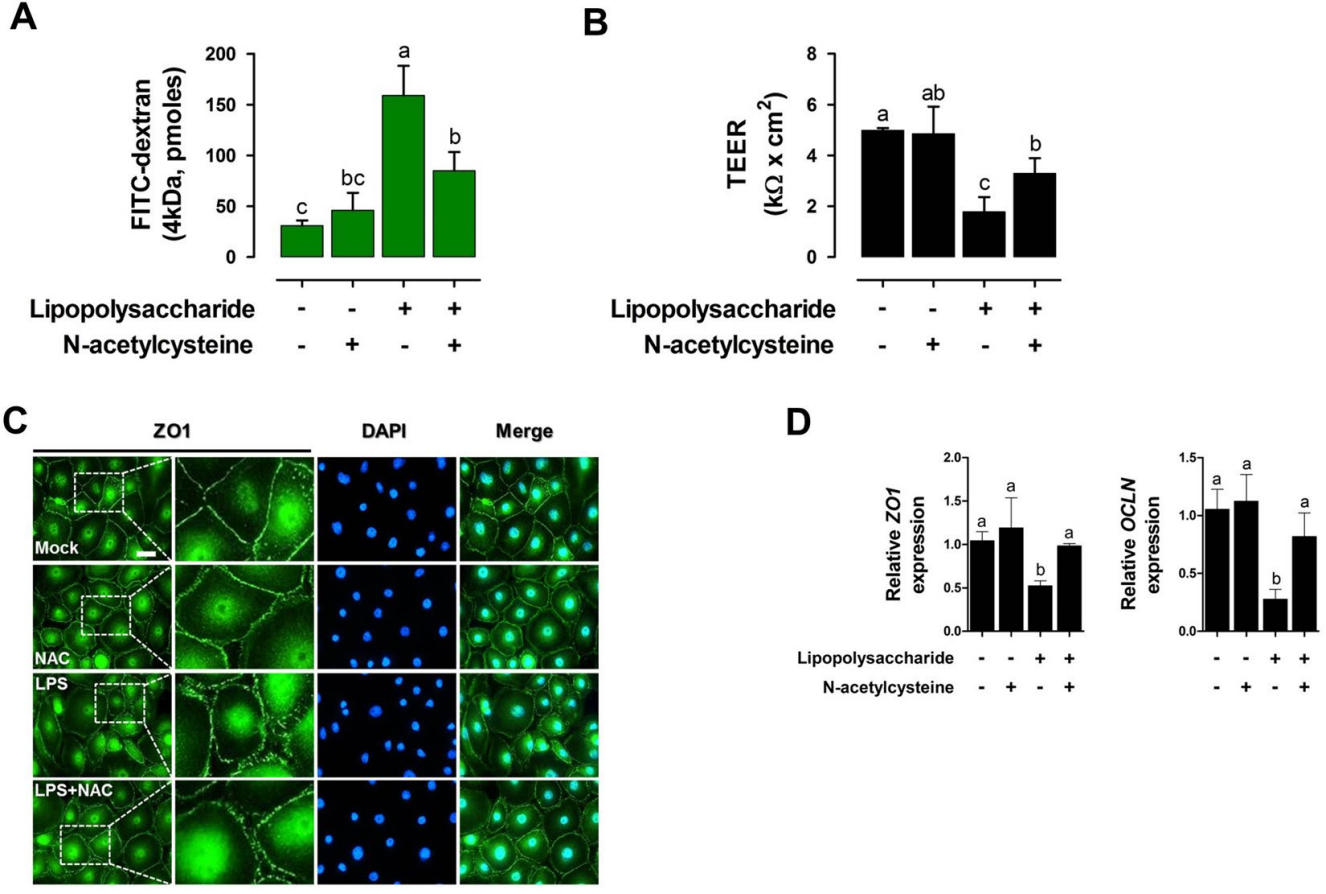
NAC treatment protects intestinal barrier function under LPS challenge. (**A**) Treatment with NAC decreased the permeability of fluorescein isothiocyanate-labelled 4 kDa dextrans (FD-4) following LPS challenge in IPEC-J2 cells (n = 3). (**B**) Treatment with NAC increased transepithelial-electrical resistance (TEER) following LPS challenge in IPEC-J2 cells (n = 3). (**C**) Immunofluorescence staining showing the effects of NAC on the expression of the tight junction protein 1 (ZO-1) in LPS-challenged IPEC-J2 cells. Nuclei were stained with DAPI. (**D**) Quantitative expression analysis of genes encoding tight junctions after NAC treatment under LPS challenge (n = 3). Relative ratio between groups was calculated by the 2^−ΔΔCt^ method and normalized by *GAPDH* housekeeping gene. Error bars indicate standard errors (SEs) of triplicate analyses and lowercase letters represent significant differences (P < 0.05).

LPS challenge increased the permeability of FD-4(P < 0.05, Fig. 6A). Under LPS challenge, treatment with NAC decreased permeability in IPEC-J2 cells (P < 0.05, Fig. 6A). LPS challenge decreased the TEER, and treatment with NAC increased TEER under LPS challenge (P < 0.05, Fig. 6B). However, treatment with NAC without LPS had no effect on permeability of FD-4 and TEER in IPEC-J2 cells compared with that in the control. The immunocytochemistry results demonstrated that ZO-1 expression level was significantly decreased in LPS challenge, and treatment with NAC in LPS-treated cells preserved the expression of ZO-1 (Fig. 6C). LPS challenge decreased the mRNA expression of ZO-1 and OCLN and treatment with NAC increased mRNA expression of ZO-1 and OCLN under LPS challenge in IPEC-J2cells (P < 0.05, Fig. 6A). However, treatment with NAC without LPS had no effect on the expression of *ZO-1* and *OCLN* in IPEC-J2 cells compared with that in the control.

### NAC regulated wound healing in LPS-induced intestinal injury

**Figure 7:**
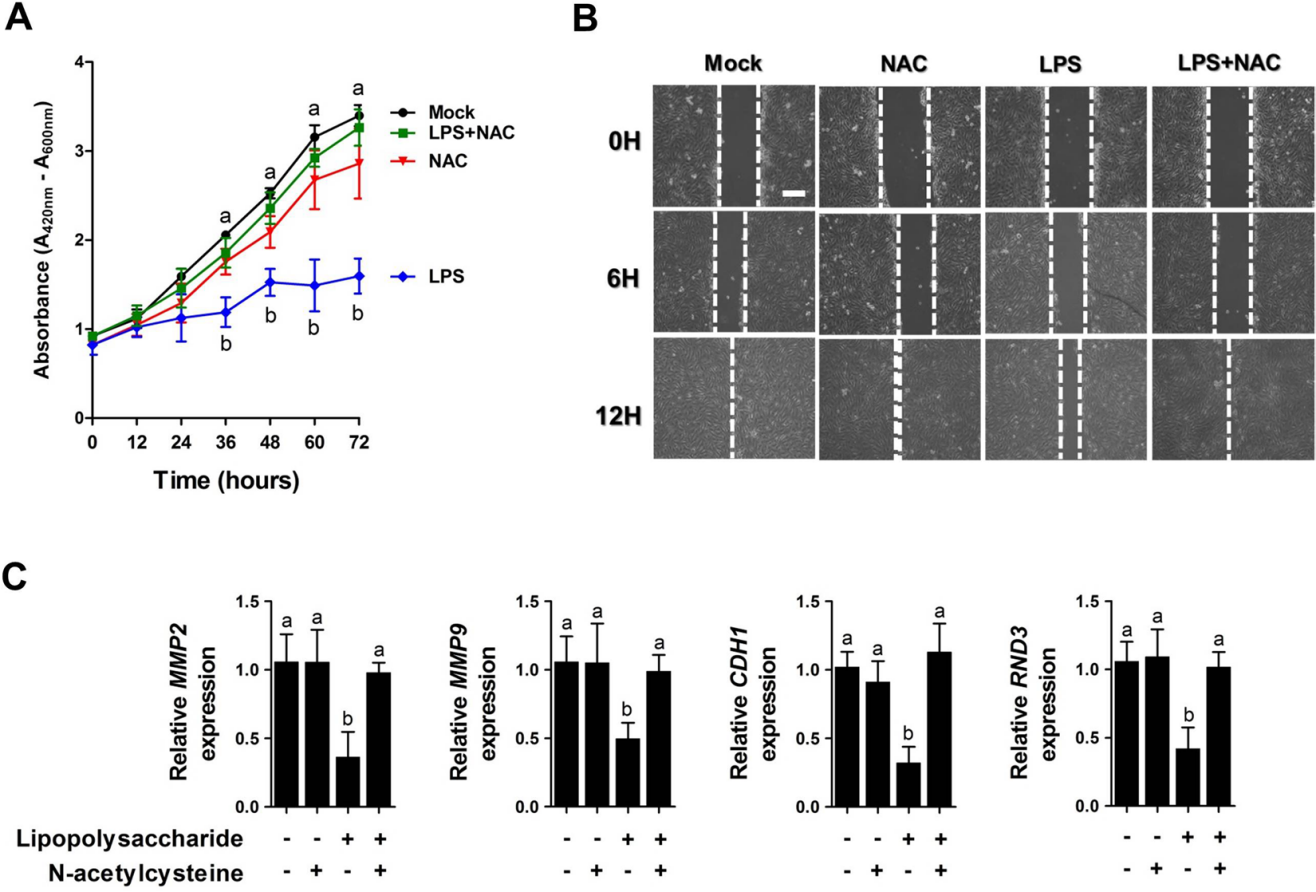
NAC regulated intestinal wound healing *in vitro*. (**A**) The number of viable cells was determined 12, 24, 36, and 48 h after treatment with NAC under LPS challenge using WST-1 assays (n = 5). (**B**) Effects of NAC on the migration of IPEC-J2 cells at 0, 6, and 12 h (n = 3). (**C**) Expression of wound healing-related genes, such as *MMP2*, *MMP9*, *CDH1*, and *RND3*, analysed with or without NAC after LPS challenge in IPEC-J2 cells using real-time polymerase chain reaction (PCR). Relative ratio between groups was calculated by the 2^−ΔΔCt^ method and normalized by *GAPDH* housekeeping gene. Error bars indicate standard errors (SEs) of triplicate analyses and lowercase letters represent significant differences (P < 0.05). *MMP*, matrix metallopeptidase; *CDH1*, cadherin 1; *RND3*, Rho family GTPase 3; MTT, 3-(4,5-dimethylthiazol-2-yl)-2,5-diphenyltetrazolium bromide; WTS, water-soluble tetrazolium.

To determine whether NAC enhances intestinal wound healing, we analysed the cell proliferation, cell migration, and expression patterns of wound healing-related genes under LPS challenge. Treatment with NAC stimulated the growth of IPEC-J2 cells under LPS challenge (P < 0.05; Fig. 7A) and significantly reduced the wound width in scratch assays under LPS challenge (Fig. 7B). Under LPS challenge, the expression of *MMP2*, *MMP4*, *CDH1*, and *RND3* was decreased compared with that in the control. However, NAC treatment significantly increased the expression of *MMP2*, *MMP4*, *CDH1*, and *RND3* under LPS challenge (P < 0.05, Fig. 7C). In contrast, treatment with NAC without LPS had no effect on the expression of wound healing-related genes in IPEC-J2 cells compared with that in the control.

## Discussion

The gastrointestinal tract is susceptible to various stresses, such as weaning in early life and challenges with pathogenic bacteria or toxins. Because intestinal dysfunction caused by various stresses is directly related to growth performance in pigs, affecting parameters such as feed intake, average daily gain, and gain/feed ratio, it is important to consider a variety of environmental factors, including diets and stressors, to maintain intestinal health^10,11^. The fact that bioactive food compounds in the diet can directly interact with genes and affect transcription factors, protein expression, and metabolite production has led to nutritional research in livestock^12^. Thus, to better understand the cellular physiology and functionality of the small intestine, in the present study we performed a transcriptomic analysis to evaluate gene expression profiles following dietary NAC treatment under LPS challenge in pigs. The gene expression profiling results revealed that NAC treatment changed the expression of genes related to immune response, inflammatory response, oxidation-reduction processes, cytokine-cytokine receptor interactions, and cytokine-mediated signalling, Toll-like receptor signalling, Jak-STAT signalling, TNF signalling pathways. Based on the gene expression profiling results, the present study further examined the physiological effects of NAC, such as those on the inflammatory response, oxidative stress, intestinal barrier function, and wound healing, under LPS challenge *in vitro*.

Our present study demonstrated that NAC treatment alleviated LPS-induced-TNF-α production in small intestinal epithelial cells. In agreement with a previous report, the adverse effects of LPS which increased the concentrations of TNF-a, interleukin-6, cortisol, and prostaglandin E2 in plasma and intestinal mucosae were attenuated by NAC supplementation^13^. Moreover, the results of the present study indicated that NAC treatment attenuated LPS-induced NF-κB expression in small intestinal epithelial cells. In the mammalian immune system, NF-κB plays a critical role as the key regulator of the innate and adaptive immune systems^14,15^. During infection, oxidative stress, or pro-inflammatory cytokine exposure, NF-κB is activated by transmembrane Toll-like receptors (TLRs) that are usually expressed in macrophages, and it plays an important role in recognizing immune-stimulating molecules and regulate the expression of inflammatory cytokines, including TNF-α, a hallmark of the cellular response leading to the activation of the innate immune system^14,16^.

Oxidative stress reflects an imbalance between the systemic manifestations of ROS produced by living organisms during normal cellular metabolism. ROS function in physiological cell processes at low concentrations; however, they induce adverse modifications to cell components, such as lipids, proteins, and DNA, at high concentrations^17^. Following the accumulation of ROS, cells attempt restore the redox balance by activating or silencing genes encoding defensive enzymes, transcription factors, structural proteins, and inflammatory cytokines, resulting in preserving cell viability, activation, proliferation, and organ function^18,19^. In the gastrointestinal tract, mucosal oxidative stress and tissue redox imbalance result in the loss of intestinal homeostasis, which affects nutrient digestion and absorption, enterocyte apoptosis, and the immune response, resulting in reduced growth performance in livestock^20^. To reduce oxidative stress in the gastrointestinal tract, many antioxidants, including NAC, have been proposed for supplementation^19^. Among antioxidants, NAC, as a useful thiol-containing antioxidant, is an effective scavenger of free radicals and contributes to the maintenance of the cellular glutathione status^21^. It has been reported that NAC tends to protect cells against oxidative stress, inhibit the development of gut injury, and reduce intestinal lipid peroxidation and the levels of free radicals, restoring the activities of antioxidant enzymes to normal levels^22,23^. In agreement with previous reports, the present study demonstrated that NAC reduced ROS levels and the expression of *SOD1*, *GCLM*, *GCLC*, and *CAT* in LPS-induced small intestinal epithelial cells.

The gastrointestinal tract has two main functions, nutrient absorption and intestinal barrier against harmful bacteria or toxins, which are carried out in the intestinal epithelium, which controls selective permeability by two major routes: transcellular and paracellular permeability^24^. Paracellular permeability prevents the transmission of harmful foreign antigens, toxins, and microorganisms, and transcellular permeability facilitates the passage of dietary nutrients, electrolytes, water, and various beneficial substances from the intestinal lumen^1^. Paracellular permeability is tightly regulated by junctional complexes that seal together adjacent cells and provide cytoskeletal anchorage in intestinal epithelial cells^2,25^. Tight junctions act as receptors or targets of bacterial virulence factors, including LPS, during the infection of a range of viral and bacterial pathogens, and disruption of tight junctions leads to increased epithelial permeability and facilitates the translocation and colonisation of pathogens into the body, resulting in diarrheal disease^26^. To induce abnormal intestinal barrier function, the present study used LPS to disrupt the tight junction between epithelial cells. The results of this study indicated that LPS challenge impaired epithelial barrier function, whereas NAC treatment offered protection against LPS-induced epithelial barrier dysfunction.

Following injury by nutrients, harmful bacteria, toxins, inflammation, and oxidative stress, the intestinal epithelium undergoes wound healing^27^. It has been reported that intestinal epithelial wound healing is modulated by various regulatory factors, including cytokines, growth factors, regulatory peptides, dietary factors, and signalling pathways^28^. To determine whether NAC as a dietary factor can modulate the intestinal wound healing process, the present study investigated the migration, proliferation, and expression of differentiation-related genes following LPS-induced intestinal epithelial cell injury and demonstrated that NAC treatment increased cell proliferation, migration, and expression of *MMP2*, *MMP4*, *CDH1*, and *RND3* under LPS-induced injury. It has been reported that specific signalling pathways including PI3K/Akt, ERK1/2 MAPK, STAT3, and NF-kB are involved in intestinal epithelial wound healing^29–32^. In addition, a previous report demonstrated that the LPS-induced adverse effects on porcine intestinal function and redox status were mitigated by NAC supplementation through the activation of multiple signalling pathways including PI3K/Akt/mTOR, EGFR, TLR4/NF-κB, AMPK, and type I IFN^33^. However, further study is required to determine the direct relationship between NAC and intestinal wound healing through signalling pathways.

It is well documented that NAC has a beneficial effect in a number of diseases including cancer, haemorrhagic cystitis, and obstructive lung disease, because of its critical role in attenuating oxidative stress, apoptosis, and mitochondrial dysfunction^9^. In agreement with previous reports, the present study analysed the beneficial effects of NAC on LPS-induced inflammation, which leads to intestinal dysfunction in small intestinal epithelial cells. Moreover, the present study evaluated the effects of NAC on intestinal functions including cytokine expression, intestinal barrier function, and intestinal wound healing under normal condition owing to the lack of reports on NAC monotherapy *in vitro*. Contrary to the result of LPS challenge, NAC had no effect on intestinal function including cytokine expression, intestinal barrier function, and intestinal wound healing in NAC treatment without LPS challenge in IPEC-J2 cells. In agreement with the present study, it has been reported that only cell proliferation, mitochondrial respiration, lactate dehydrogenase concentration, and apoptosis were not affected by NAC in small intestinal epithelial cells^34^. The function of NAC might be inadequate, but it can be effective in special condition such as inflammation.

In conclusion, we found that dietary supplementation with NAC altered the expression of 959 genes related to immune response, inflammatory response, oxidation-reduction processes, cytokine-cytokine receptor interactions, and cytokine-mediated signalling, Toll-like receptor signalling, Jak-STAT signalling, and TNF signalling pathways under LPS challenge in the small intestine of pigs. To confirm the gene expression profiling results, we analysed the expression pattern of the top 10 altered genes and confirmed that NAC markedly stimulated the expression of *HMGCS3* and *LDHC* in IPEC-J2 cells. The present study revealed that pre-treatment with NAC significantly reduced TNF-α level and the expression of *NF-κB*, *TNF-α*, *IFN-γ*, and *IL-6* in LPS-induced IPEC-J2 cells. NAC treatment also significantly reduced oxidative stress, as indicated by reduced DCF levels and increased expression of *SOD1*, *GCLM*, *GCLC*, and *CAT* in LPS-induced IPEC-J2 cells. Furthermore, the present study revealed that NAC treatment alleviated intestinal barrier function and wound healing. Collectively, these data indicate that NAC can be used as a feed additive to enhance intestinal health by modulating intestinal inflammation, permeability, and wound healing under LPS-induced dysfunction, including inflammation, oxidative stress, and injury.

## Materials and Methods

The animal care and experimental protocols of the present study were approved by the Animal Care, and Use Committee of Dankook University and all methods were performed in accordance with the relevant guidelines and regulations.

### Experimental animals and diet

To evaluate the effects of NAC, a feeding trial was performed using 15 miniature pigs [MK strain, (Duroc × Yorkshire) × (Pot Valley × Berkshire) × Yucatan] with an average initial body weight of 21.34 ± 0.51 kg during a 21-d period. Corn and soybean meal-based diets were used as the feed and experimental treatments were divided into three groups: control diet + saline challenge, control diet + LPS challenge, and control diet with 500 mg/kg NAC + LPS challenge.

LPS challenge assay was performed according to our previous report^35^. Briefly, all pigs were injected with LPS (serotype O55:B5; Sigma-Aldrich) at 0.01% (50 mg/kg) of the body weight on day 21 of the feeding trial.

### Expression profiling analysis

During the feeding trial, small intestines were collected from all pigs, pooled, and stored at −80 °C. Total RNA was extracted and qualified using an Agilent 2100 bioanalyzer with an RNA 6000 Nano Chip (Agilent Technologies, Amstelveen, The Netherlands). For library preparation and sequencing, library construction was performed using a SENSE 3′ mRNA-Seq Library Prep Kit (Lexogen, Inc., Austria) according to the manufacturer’s instructions. In brief, each 500 ng sample of total RNA was prepared, and an oligo-dT primer containing an Illumina-compatible sequence at its 5′ end was hybridised to the RNA; reverse transcription was then performed. After degradation of the RNA template, second-strand synthesis was initiated by a random primer containing an Illumina-compatible linker sequence at its 5′ end. The double-stranded library was purified using magnetic beads to remove all reaction components. The library was amplified to add the complete adapter sequences required for cluster generation. The completed library was purified from PCR components. High-throughput sequencing was performed by single-end 75 sequencing using a NextSeq. 500 instrument (Illumina, Inc., USA).

### Culture condition and ELISA

The IPEC-J2 cells were cultured according to our previous report^36^. Culture of IPEC-J2 cells was performed in DMEM/F-12 (Gibco Life Technologies, Grand Island, NY, USA) supplemented with 5% FBS, 1% insulin-transferrin-selenium-X, and 1% (v/v) penicillin-streptomycin mixture. All cell cultures were maintained at 37 °C in a humidified atmosphere of 5% CO_2_. Various concentrations of NAC were used to treat IPEC-J2 cells for 24 h before LPS challenge. For ELISA assay, culture media were collected 4 h after LPS treatment and were centrifuged. TNF-α expression was evaluated using ELISA kit (Thermo Scientific, Waltham, MA, USA).

### Quantitative real-time polymerase chain reaction

Total RNA (1 µg) was extracted from IPEC-J2 cells using TRIzol reagent (Invitrogen, Carlsbad, CA, USA) and used for cDNAs synthesis with a Maxima First-strand cDNA Synthesis Kit (Life Technologies). The primers for qRT-PCR for each gene transcript were designed using Primer3 (http://frodo.wi.mit.edu/; Supplemental Table 9). qRT-PCR was performed using a 7500 Fast Real-time PCR System (Applied Biosystems) that was programmed to subject the samples to denaturation at 94 °C for 3 min, followed by 40 cycles at 94 °C for 30 s and 59–61 °C for 30 s, and 72 °C for 30 s. All gene quantitation were performed using the 2^−ΔΔCt^ method^37^.

### Intracellular ROS, transepithelial electrical resistance, and intestinal permeability analysis

The average density of intracellular ROS was evaluated in cells loaded with the redox-sensitive dye dichloro-dihydro-fluorescein diacetate (Molecular Probes DCFH-DA; Thermo Fisher Scientific, Inc.). IPEC-J2 cells, with or without NAC pre-treatment, were seeded in 96-well culture plates, treated for 1 h with LPS, washed twice with PBS, stained with 100 *µ*M DCFH-DA in the dark for 30 min, and harvested. The cells were then dissolved with 1% Triton X-100 (Sigma-Aldrich). Fluorescence was measured at excitation and emission wavelengths of 485 and 530 nm, respectively, using a fluorescence spectrometer (BioTek Instruments, Winooski, VT, USA).

Confluent monolayers of IPEC-J2 cells were grown in 24-well Corning Transwell chambers (polycarbonate membrane, filter pore size 0.4 μm, area 0.33 cm^2^; Costar) and then treated with or without NAC for 24 h. IPEC-J2 cells were treated with LPS (1 μg/μL) for 1 h. TEER was measured using an epithelial volt-ohm meter (World Precision Instruments, Sarasota, FL, USA). TEER values were calculated by subtracting the blank filter (90 Ω) and by multiplying the surface area of the filter. All measurements were performed on a minimum of triplicate wells.

NAC was treated with or without NAC for 24 h before LPS challenge. After LPS challenge, paracellular permeability was measured using 1 mg/mL FD-4 (Sigma-Aldrich). FD-4 was dissolved in culture medium and loaded on an apical chamber at 1 mg/mL. After 18 h of incubation, the amount of fluorescence on the basolateral side was measured using a flour-spectrophotometer (Ex/Em: 490/520 nm).

### Immunofluorescence

For immunofluorescence, IPEC-J2 cell monolayers grown on glass coverslips were fixed in 4% paraformaldehyde. Following blocking with 2% bovine serum albumin in PBS, the cells were incubated with primary antibodies used at a dilution of 1:100 (ZO-1) overnight. After repeated washing, fluorophore-conjugated secondary antibodies (Alexa Fluor 488) were applied for 1 h at room temperature in the dark, and the cells were washed again. Mounting was performed using VECTASHIELD Antifade Mounting Medium with DAPI (Vector Laboratories, Burlingame, CA, USA), and images were collected using a fluorescence microscope. Fluorescence intensities were quantified using the threshold function of ImageJ software.

### Cell proliferation and migration assays

IPEC-J2 cells were seeded in 96-well plates at a density of 1 × 10^4^ cells/well. The cell proliferation reagent WST-1 (Roche Applied Science, Indianapolis, IN, USA) was added to each well. The absorbance of the dye after incubation was measured at a wavelength of 450 nm with background subtraction at 690 nm using a BioTek Synergy HTTR microplate reader (BioTek Instruments, Winooski, VT, USA). IPEC-J2 cells were cultured with or without NAC in 60-mm culture dishes until reaching confluence. Subsequently, straight scratches were made with a p200 pipette tip across the 60-mm culture dish, simulating a wound. Photographs were acquired at different intervals, as indicated in the figure legends.

### Statistical analysis

PROC-GLM procedure was used to determine the biological significance of differences between the groups using SAS program. The individual pigs were considered as the experimental unit. A P-value of less than 0.05 indicated statistical significance. Significant differences between control and treatment groups are indicated as ***P < 0.001, **P < 0.01, and *P < 0.05. Significant differences between groups were compared by Duncan multiple range tests.

## Supplementary information

Supplemental information

## References

[1] Capaldo CT, Powell DN, Kalman D (2017). Layered defense: how mucus and tight junctions seal the intestinal barrier. J Mol Med (Berl).

[2] Giepmans BN, van Ijzendoorn SC (2009). Epithelial cell-cell junctions and plasma membrane domains. Biochimica et biophysica acta.

[3] Zhu LH, Zhao KL, Chen XL, Xu JX (2012). Impact of weaning and an antioxidant blend on intestinal barrier function and antioxidant status in pigs. Journal of animal science.

[4] Wijtten PJ, van der Meulen J, Verstegen MW (2011). Intestinal barrier function and absorption in pigs after weaning: a review. The British journal of nutrition.

[5] Wu G, Fang YZ, Yang S, Lupton JR, Turner ND (2004). Glutathione metabolism and its implications for health. The Journal of nutrition.

[6] Wu G (2009). Amino acids: metabolism, functions, and nutrition. Amino acids.

[7] Fishbane S, Durham JH, Marzo K, Rudnick M (2004). N-acetylcysteine in the prevention of radiocontrast-induced nephropathy. Journal of the American Society of Nephrology: JASN.

[8] Zafarullah M, Li WQ, Sylvester J, Ahmad M (2003). Molecular mechanisms of N-acetylcysteine actions. Cellular and molecular life sciences: CMLS.

[9] Hou Y, Wang L, Yi D, Wu G (2015). N-acetylcysteine and intestinal health: a focus on its mechanism of action. Front Biosci (Landmark Ed).

[10] Camilleri M, Madsen K, Spiller R, Greenwood-Van Meerveld B, Verne GN (2012). Intestinal barrier function in health and gastrointestinal disease. Neurogastroenterology and motility: the official journal of the European Gastrointestinal Motility Society.

[11] Zhang D (2017). Gene expression profile changes in the jejunum of weaned piglets after oral administration of Lactobacillus or an antibiotic. Scientific reports.

[12] Garcia-Canas V, Simo C, Leon C, Cifuentes A (2010). Advances in Nutrigenomics research: novel and future analytical approaches to investigate the biological activity of natural compounds and food functions. Journal of pharmaceutical and biomedical analysis.

[13] Hou Y (2012). Protective effects of N-acetylcysteine on intestinal functions of piglets challenged with lipopolysaccharide. Amino acids.

[14] Akira S (2006). TLR signaling. Current topics in microbiology and immunology.

[15] Carpenter S, O’Neill LAJ (2007). How important are Toll-like receptors for antimicrobial responses?. Cellular microbiology.

[16] Baeuerle PA, Henkel T (1994). Function and activation of NF-κB in the immune system. Annual review of immunology.

[17] Valko M, Rhodes CJ, Moncol J, Izakovic M, Mazur M (2006). Free radicals, metals and antioxidants in oxidative stress-induced cancer. Chemico-biological interactions.

[18] Dalton TP, Shertzer HG, Puga A (1999). Regulation of gene expression by reactive oxygen. Annual review of pharmacology and toxicology.

[19] Birben E, Sahiner UM, Sackesen C, Erzurum S, Kalayci O (2012). Oxidative stress and antioxidant defense. The World Allergy Organization journal.

[20] Circu ML, Aw TY (2012). Intestinal redox biology and oxidative stress. Seminars in cell & developmental biology.

[21] Kerksick C, Willoughby D (2005). The antioxidant role of glutathione and N-acetyl-cysteine supplements and exercise-induced oxidative stress. Journal of the International Society of Sports Nutrition.

[22] Koivusalo A (2002). Intraluminal casein model of necrotizing enterocolitis for assessment of mucosal destruction, bacterial translocation, and the effects of allopurinol and N-acetylcysteine. Pediatric surgery international.

[23] Xu CC (2014). Regulation of N-acetyl cysteine on gut redox status and major microbiota in weaned piglets. Journal of animal science.

[24] Groschwitz, K. R. & Hogan, S. P. Intestinal barrier function: molecular regulation and disease pathogenesis. *The Journal of allergy and clinical immunology* **124**, 3–20, quiz 21–22, 10.1016/j.jaci.2009.05.038 (2009). 10.1016/j.jaci.2009.05.038 PMC4266989 19560575

[25] Laukoetter MG, Bruewer M, Nusrat A (2006). Regulation of the intestinal epithelial barrier by the apical junctional complex. Current opinion in gastroenterology.

[26] Konig J (2016). Human Intestinal Barrier Function in Health and Disease. Clinical and translational gastroenterology.

[27] Moyer RA, Wendt MK, Johanesen PA, Turner JR, Dwinell MB (2007). Rho activation regulates CXCL12 chemokine stimulated actin rearrangement and restitution in model intestinal epithelia. Laboratory investigation; a journal of technical methods and pathology.

[28] Iizuka M, Konno S (2011). Wound healing of intestinal epithelial cells. World journal of gastroenterology.

[29] El-Assal ON, Besner GE (2005). HB-EGF enhances restitution after intestinal ischemia/reperfusion via PI3K/Akt and MEK/ERK1/2 activation. Gastroenterology.

[30] Sheng H, Shao J, Townsend CM, Evers BM (2003). Phosphatidylinositol 3-kinase mediates proliferative signals in intestinal epithelial cells. Gut.

[31] Spehlmann ME, Eckmann L (2009). Nuclear factor-kappa B in intestinal protection and destruction. Current opinion in gastroenterology.

[32] Pickert G (2009). STAT3 links IL-22 signaling in intestinal epithelial cells to mucosal wound healing. The Journal of experimental medicine.

[33] Yi D (2017). N-Acetylcysteine improves intestinal function in lipopolysaccharides-challenged piglets through multiple signaling pathways. Amino acids.

[34] Xiao H (2016). N-Acetyl-L-cysteine Protects the Enterocyte against Oxidative Damage by Modulation of Mitochondrial Function. Mediators of inflammation.

[35] Lee SI, Kim HS, Koo JM, Kim IH (2016). Lactobacillus acidophilus modulates inflammatory activity by regulating the TLR4 and NF-kappaB expression in porcine peripheral blood mononuclear cells after lipopolysaccharide challenge. The British journal of nutrition.

[36] Lee SI, Kim IH (2018). Nucleotide-mediated SPDEF modulates TFF3-mediated wound healing and intestinal barrier function during the weaning process. Scientific reports.

[37] Livak KJ, Schmittgen TD (2001). Analysis of relative gene expression data using real-time quantitative PCR and the 2(−Delta Delta C(T)) Method. Methods.

