# Supplementary material for: N-acetylcysteine modulates lipopolysaccharide-induced intestinal dysfunction
Source: Sci Rep. 2019 Jan 30;9:1004. doi: 10.1038/s41598-018-37296-x (PMC6353963; doi:10.1038/s41598-018-37296-x)
Supplement: Supplementary file 1 — Supplemental information [file 41598_2018_37296_MOESM1_ESM.pdf]

N-acetylcysteine modulates lipopolysaccharide-induced intestinal dysfunction

Sang In Lee[1]and Kyung Soo Kang[2]*

Supplemental figure 1

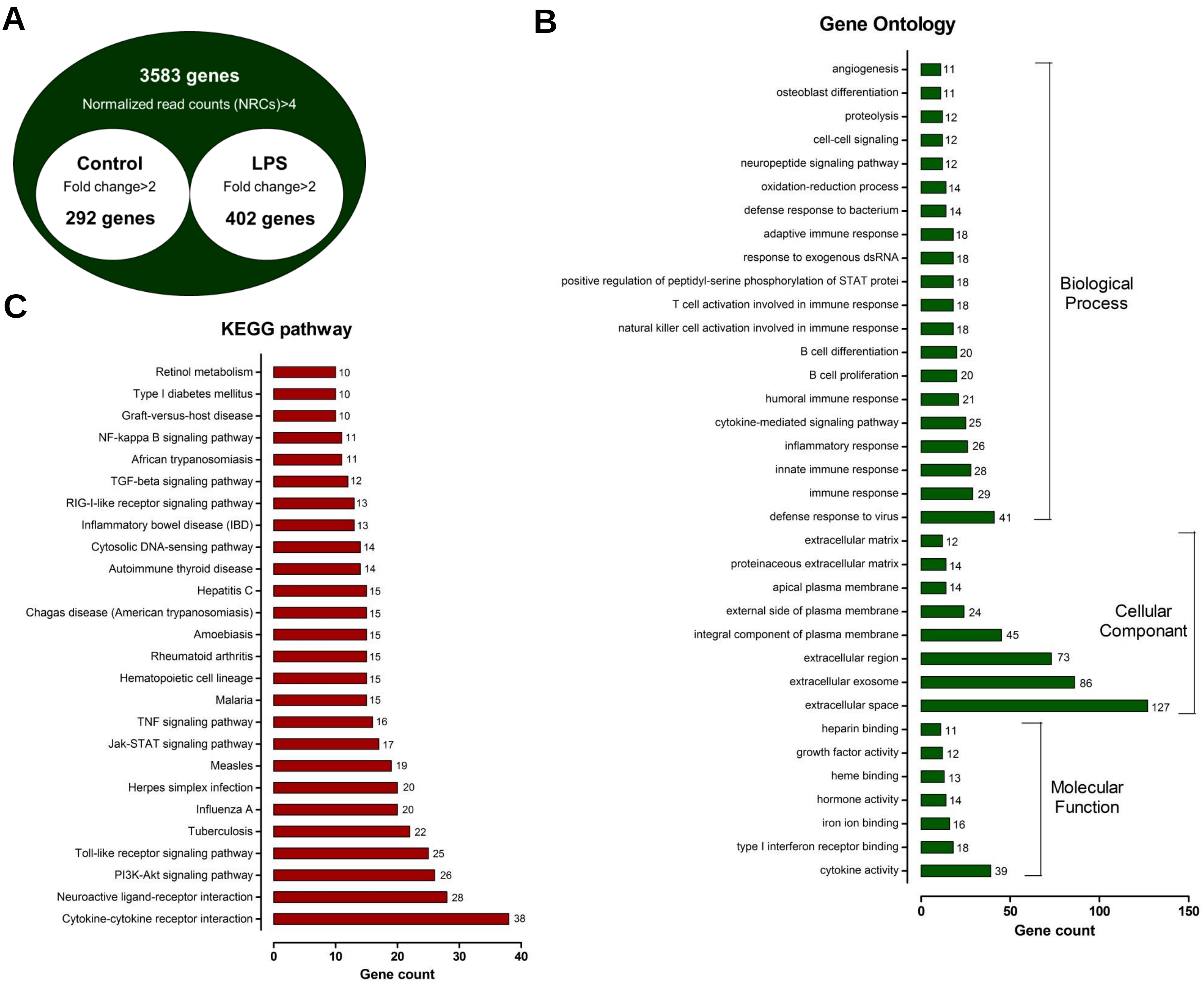

Figure legend

Supplemental Figure 1. Gene expression profiling of the small intestine after dietary treatment with or without LPS challenge (a) Venn diagram illustrations of genes found to be differentially expressed with or without LPS challenge in the small intestine. The common genes from the RNAseq experiment were upregulated at least 2-fold ($P < 0.05$). (b) Gene Ontology (GO) enrichment analysis of differentially expressed genes. GO terms assigned to biological process, cellular component, and molecular functions. (c) Kyoto Encyclopedia of Genes and Genomes (KEGG) pathway classification enrichment analysis of differentially expressed genes.

Supplemental table 1. Biological process in differentially expressed genes (LPS vs LPS+NAC)

Term	Count	P-Value	Genes
ssc04060:Cytokine-cytokine receptor interaction	39	2.26E-08	IL1R2, CSF2, CCL2, BMPR2, IL-6, CD70, CCL5, CCL4, CCL27, IFN-ALPHA-8, IFN-ALPHA-13, CCL25, IFNA1, IFN-ALPHA-15, IFN-ALPHA-9, IL12RB1, CCL20, IFNG, LTB, IL1A, LTA, GHR, IL6, TNFSF4, IL7, CCL19, LOC100153661, CCR9, ACVR2A, TNFSF10, ACVR2B, CCR7, IL1B2, IL1B1, IL12A, EPOR, BMPR1B, BMP7, BMPR1A
ssc05168:Herpes simplex infection	33	1.79E-07	RNASEL, IFIH1, CCL2, TLR3, IL-6, OAS1, CCL5, TLR9, IFN-ALPHA-8, IFN-ALPHA-13, CASP3, IFNA1, IFN-ALPHA-15, IFN-ALPHA-9, PKR, IFNG, CASP8, TRAF6, CHUK, LTA, CDK1, IL6, SKP2, ARNTL, STAT1, LOC100153661, STAT2, IFIT1, IL1B2, IRF7, IL1B1, IL12A, JAK2
ssc05162:Measles	30	4.00E-09	IFIH1, IL-6, OAS1, TLR9, IFN-ALPHA-8, IRAK4, IFN-ALPHA-13, IFNA1, IFN-ALPHA-15, IFN-ALPHA-9, PKR, CD46, IFNG, TRAF6, IL1A, CHUK, AKT3, PIK3CG, IL6, CD3E, STAT1, LOC100153661, STAT2, TNFSF10, IL1B2, FCGR2B, IRF7, IL1B1, IL12A, JAK2
ssc05164:Influenza A	29	2.85E-06	RNASEL, IFIH1, CCL2, TLR3, IL-6, OAS1, CCL5, IFN-ALPHA-8, IRAK4, IFN-ALPHA-13, IFNA1, IFN-ALPHA-15, IFN-ALPHA-9, PKR, IFNG, CASP1, AKT3, IL1A, PIK3CG, IL6, STAT1, LOC100153661, STAT2, TNFSF10, IL1B2, IRF7, IL1B1, IL12A, JAK2
ssc04620:Toll-like receptor signaling pathway	27	6.99E-10	IL-6, PMAP-36, TLR9, IRAK4, IFN-ALPHA-8, IFN-ALPHA-13, CASP3, IFNA1, IFN-ALPHA-15, IFN-ALPHA-9, IFNG, CASP8, NOS2, TRAF6, IL1A, AKT3, IL6, CREB1, STAT1, LOC100153661, CASP10, FCGR2B, IL1B2, IL1B1, IL12A, JAK2, CD14

ssc05160:Hepatitis C	27	1.19E-07	PPARA, RNASEL, OCLN, CLDN5, LOC396566, TLR3, OAS1, CLDN10, CLDN14, IFN-ALPHA-8, IFN-ALPHA-13, IFNA1, IFN-ALPHA-15, IFN-ALPHA-9, PKR, TRAF6, CHUK, AKT3, PIK3CG, CLDN22, CLDN20, STAT1, LOC100153661, STAT2, IFIT1, IRF7, CLDN1
ssc05152:Tuberculosis	27	5.24E-05	TLR3, IL-6, TLR5, CCL5, CCL4, TLR8, TLR9, IRAK4, IFN-ALPHA-8, IFN-ALPHA-13, IFNA1, IFN-ALPHA-15, IFN-ALPHA-9, CASP8, TRAF6, CHUK, AKT3, PIK3CG, IL6, STAT1, LOC100153661, CD86, IL1B2, IRF7, IL1B1, IL12A, CD14
ssc03320:PPAR signaling pathway	26	1.52E-13	ACOX1, PPARA, ATP1B1, DRD2, GLI1, EDNRA, ATP2B1, HTR1B, BDNF, ADCY10, HTR1D, AKT3, PIK3CG, PLD1, CREB1, ATP1A2, TNNI3, SSTR2, NPY, CHRM2, CHRM1, PLN, GHRL, GHSR, LIPE, GLP1R
ssc04630:Jak-STAT signaling pathway	26	3.05E-06	CSF2, IL-6, STAT6, IFN-ALPHA-8, IFN-ALPHA-13, IFNA1, IFN-ALPHA-9, IFN-ALPHA-15, IL12RB1, IFNG, AKT3, GHR, IL13RA2, PIK3CG, IL6, IL7, SOCS7, SOCS4, SOCS5, STAT1, LOC100153661, STAT2, IL12A, JAK2, EPOR
ssc04024:cAMP signaling pathway	26	0.001019931	ACOX1, PPARA, AQP7, MMP1, APOA2, APOA1, PLIN1, APOC3, APOA5, CYP4A24, ACSL3, PLTP, ACSL5, ANGPTL4, ACADM, PCK2, CPT1A, PCK1, CD36, FABP3, FABP1, FABP2, GK, FABP7, SCP2, FABP6
ssc05161:Hepatitis B	25	3.67E-05	PIK3CG, IL6, IFIH1, EGR2, CREB1, TLR3, IL-6, RB1, LOC100153661, STAT1, STAT2, IFN-ALPHA-8, STAT6, CASP10, IFN-ALPHA-13, CASP3, IFNA1, IFN-ALPHA-9, IFN-ALPHA-15, IRF7, CASP8, CCNA2, AKT3, CHUK
ssc04062:Chemokine signaling pathway	22	0.004054441	AMCF-II, CCL1, PIK3CG, CCL2, CCL19, STAT1, CCL5, CCL4, CCL27, CCL17, STAT2, CCR9, CCL25, CCR7, CCL14, CCL20, RASGRP2, JAK2, GNB3, CRK, AKT3, CHUK

ssc04640:Hematopoietic cell lineage	20	5.65E-07	PIK3CG, IL6, CCL2, CD3E, GNA11, IL-6, CCL5, TLR9, IRAK4, ACE, IL1B2, GNAQ, CASP8, IFNG, IL1B1, IL12A, NOS2, TRAF6, AKT3, CHUK
ssc05142:Chagas disease (American trypanosomiasis)	20	5.10E-05	IL1R2, CSF2, IL6, IL7, CD3E, CD8B, FCER2, CD1.1, IL-6, ANPEP, GP9, CD36, CD19, IL1B2, CD59, IL1B1, MS4A1, EPOR, CD14, IL1A
ssc04668:TNF signaling pathway	19	1.86E-04	PIK3CG, CSF2, IL6, CCL2, PTGS2, CREB1, IL-6, MMP3, CCL5, CASP10, CASP3, IL1B2, CCL20, CASP8, IL1B1, SELE, LTA, AKT3, CHUK
ssc05323:Rheumatoid arthritis	18	3.06E-05	PIK3CG, IL1R2, CSF2, IL6, GNA11, IL-6, ACTN2, C8A, C8B, CASP3, IL1B2, GNAQ, IL1B1, IFNG, IL12A, SERPINB10, NOS2, CD14
ssc05146:Amoebiasis	18	3.55E-04	AMCF-II, CSF2, IL6, CCL2, IL-6, MMP3, CCL5, MMP1, ATP6V1C1, ATP6V1A, CD86, IL1B2, CCL20, IL1B1, IFNG, ANGPT1, LTB, IL1A
ssc04152:AMPK signaling pathway	17	0.003607132	PIK3CG, PRKAG3, CREB1, PRKAB2, PFKFB1, ADIPOR2, FBP2, PCK2, CPT1A, PCK1, CD36, GYS2, ADRA1A, PPP2R5E, CCNA2, LIPE, AKT3
ssc04920:Adipocytokine signaling pathway	16	3.96E-05	PRKAG3, PPARA, PRKAB2, ADIPOR2, ACACB, PCK2, CPT1A, PCK1, CD36, NPY, JAK2, AGRP, ACSL3, AKT3, CHUK, ACSL5
ssc00830:Retinol metabolism	15	5.90E-06	PRKAG3, LDHC, PPARA, CREB1, PFKFB1, PRKAB2, ACACB, PCK2, CPT1A, PCK1, GCG, GNAQ, SLC2A2, GYS2, AKT3
ssc04623:Cytosolic DNA-sensing pathway	15	2.68E-05	KCNMA1, TRPC1, ATP1B1, PNLIPRP1, CLCA1, ATP1A2, SCTR, ATP2B1, GNAQ, ATP2A1, PLA2G12B, CELA2A, SLC4A4, PLA2G2D, PLA2G2F
ssc04972:Pancreatic secretion	15	0.001204216	IL6, IL-6, LOC100153661, CCL5, CCL4, IFN-ALPHA-8, IFN-ALPHA-13, IFNA1, IFN-ALPHA-9, IFN-ALPHA-15, IL1B2, IRF7, IL1B1, CASP1, CHUK

ssc04922:Glucagon signaling pathway	15	0.003169166	RDH12, UGT1A6, LRAT, CYP1A1, CYP2C42, UGT2B31, AOX1, AOX2, SDR16C5, CYP2A19, CYP1A2, RDH16, CYP4A24, CYP2B22, CYP2C49
ssc05204:Chemical carcinogenesis	13	4.00E-04	AMCF-II, IL6, IL-6, IRAK4, CASP3, IL1B2, IL1B1, IL12A, NOS2, CASP1, TRAF6, IL1A, CD14
ssc05321:Inflammatory bowel disease (IBD)	13	4.70E-04	IFIH1, LOC100153661, IFN-ALPHA-8, IFN-ALPHA-13, CASP10, IFNA1, IFN-ALPHA-15, IFN-ALPHA-9, IRF7, CASP8, IL12A, TRAF6, CHUK
ssc00590:Arachidonic acid metabolism	13	5.49E-04	GPX2, PLA2G16, CYP2J34, PTGS2, CYP2C42, PLA2G12B, EPHX2, GGT1, PLA2G2D, CYP2B22, CYP4A24, CYP2C49, PLA2G2F
ssc04622:RIG-I-like receptor signaling pathway	13	0.001295649	STAT6, IL6, IL12RB1, IL1B2, IFNG, IL1B1, IL12A, IL-6, TLR5, FOXP3, STAT1, IL1A
ssc05133:Pertussis	13	0.002433038	GSTA1, GSTA2, CYP1A1, PTGS2, CYP2C42, CYP1A2, CYP2B22, UGT1A6, LOC780435, LOC100514231, UGT2B31, GSTO1, CYP2C49
ssc00982:Drug metabolism - cytochrome P450	12	2.41E-04	KCNMA1, ATP2B1, KCNN4, ATP1B1, GNAQ, LYZ, AQP5, ADRA1A, ATP1A2, PMAP-36, DMBT1
ssc04970:Salivary secretion	12	0.007941387	GSTA1, UGT1A6, GSTA2, LOC780435, LOC100514231, FMO1, MAOA, UGT2B31, AOX1, AOX2, CYP1A2, GSTO1
ssc05143:African trypanosomiasis	11	3.95E-05	IRAK4, IL1B2, PTGS2, IFNG, IL1B1, IL12A, JAK2, NOS2, TRAF6, STAT1, IL1A
ssc05140:Leishmaniasis	11	0.006272287	CASP3, IL6, IL1B2, IL1B1, CASP8, IL12A, IL-6, TLR5, SAR1B, CASP1, CD14
ssc05134:Legionellosis	11	0.006272287	IL6, APOA1, GNAQ, IL1B2, THOP1, IFNG, IL1B1, IL12A, IL-6, SELE, TLR9
ssc04975:Fat digestion and absorption	10	2.36E-04	IL6, CCL2, CD36, IL1B2, IFNG, IL1B1, IL12A, IL-6, SELE, TLR9
ssc04621:NOD-like receptor signaling pathway	10	0.004455668	IL6, CCL2, IL1B2, IL1B1, CASP8, IL-6, CCL5, TRAF6, CASP1, CHUK
ssc05144:Malaria	10	0.005829896	APOA4, PNLIPRP1, CD36, APOA1, PLA2G12B, FABP1, FABP2, PLA2G2D, PLA2G2F, MTTP

Supplemental table 2. Molecular function in differentially expressed genes (LPS vs LPS+NAC)

Term	Count	P-Value	Genes
GO:0005615~extracellular space	128	4.62E-30	EDN3, S100A8, S100A9, GDF5, POSTN, VTN, TTR, APOA1, IFNG, IFN-OMEGA-6, MS4A1, IFN-OMEGA-4, IFN-DELTA-11, IFN-OMEGA-5, IFN-DELTA-10, IFN-OMEGA-2, MUC13, PPAG3, IL1A, LOC100157354, AMY2, WNT10B, CLCA1, ADAMTS20, BPIFA1, F8, MFGE8, LOC100153661, CBLN3, F3, STC1, AMCF-II, XDH, CCL1, BPIFB1, CCL2, BMPR2, CD70, GAST, CCL5, IFN-ALPHA-17, IL34, CCL4, IFN-ALPHA-16, IFN-ALPHA-13, IFNA1, IFN-ALPHA-14, ACE, IFN-ALPHA-15, ALB, SFTPD, ANGPTL1, SERPINA3-2, AGRP, PLTP, TNFSF10, IL1B2, NPY, SFRP2, CD59, IL1B1, AREG, BMP7, BMP5, IGFBP6, UFBP, IL-6, ANPEP, IFN-ALPHA-8, NPG4, IFN-ALPHA-9, SERPINA7, IFN-ALPHA-4, HAMP, IFN-ALPHA-5, TGFA, LTF, ANGPT1, LTB, DPEP1, LTA, HYAL1, IFN-DELTA-9, ZP3, CILP, IFN-DELTA-5, LYZ, IFN-DELTA-6, IFN-DELTA-7, INHA, IFN-DELTA-1, IFN-DELTA-2, IFN-DELTA-3, IFN-DELTA-4, PROC, INHBA, IL12A, SLPI, GHRL, CSF2, NDP, CCL25, CCL20, C1QTNF2, SERPINB10, KRT2, PTX3, LOC100156936, IL6, TNFSF4, IL7, ICA, FBN1, CCL19, PAG6, CELA1, LOC100155760, CCL17, GCG, AFP, DKK1, CCL14, ACE2, GDF11, CELA2A, IGFBP1, GKN1

GO:0070062~extracellular exosome	121	4.22E-04	LDHC, ATP1B1, S100A8, CHMP4C, GNA11, S100A9, AQP5, RPLP2, ENPEP, AQP2, ST3GAL1, APOA4, TTR, APOA2, ASPA, GOT1, APOA1, APOE, CD46, MS4A1, APOM, MUC13, RAB27A, ACAA2, F11R, MYO6, MFGE8, ACTN2, FBP2, NPC1, F3, ABAT, BPIFB1, FCER2, CD70, GALM, ACE, RPS28, STX12, HNMT, ALB, APOC3, ANGPTL1, AGRP, GPD1, CACNA2D1, EPHX2, S100A10, LGALS8, CD5L, DNASE1, TNFSF10, CD59, IL1B1, CD79B, FABP1, SLC46A3, STEAP4, IGFBP6, DUOX2, ANPEP, SDCBP2, GPX2, ACTR2, SERPINA7, ITIH4, LTF, ANGPT1, CAT, TUBB1, RHOF, DPP4, DPEP1, HYAL1, CDK1, CILP, LYZ, CYB5A, MAN1A1, DECR1, ATP6V1C1, C8A, MTMR11, ATP6V1A, C8B, CD86, CTH, PSME2, CLIC5, SCIN, CYBRD1, CA4, SLPI, GNB3, CAPS, MAL2, C6, APLP2, TAC3, TMEM33, FAT1, KRT2, PAFAH1B1, HRG, ETFA, DLST, LIPA, FBN1, HOGA1, KLK1, PCK2, ANXA4, PCK1, SDHB, CD19, KRT74, IRF6, AOX1, ACE2, MAT2B, CP
GO:0005576~extracellular region	85	2.64E-26	CGA, WAP-3, WAP-1, IGFBP6, FST, IL-6, SAL1, PMAP-36, CDSN, ST3GAL1, NPG1, BDNF, NPG3, NPG4, E4, ITIH4, LTF, ITIH2, ANGPT1, GUCA2A, GUCA2B, CASP1, GHR, COL10A1, CRISP2, LYZ, PMAP-23, MFGE8, F7, FGF21, INHA, PG-2, PROC, INHBA, CHGA, NPC1, IL12A, GHRL, PLA2G2D, PLA2G2F, PR39, C14H10ORF99, AWN, GAST, OAS1, TRH, CCL4, LEAP2, CCL27, TAC3, CCL25, PRRG4, ZPBP, C1QTNF3, FGB, PLA2G12B, SCG5, AGRP, IL6, PNLIPRP1, LOC396757, LOC396756, IL7, LOC396897, FBN1, CRSP3, PTGFR, AGER, LCN2, SFRP5, DNASE1, LOC692190, SPAI-2, DKK1, NPY, CD59, NPW, PON1, GDF11, ADM5, ADM2, IGFBP1, BMP7, BMP5, DMBT1
GO:0005789~endoplasmic reticulum membrane	22	0.009538405	HSD3B1, CYP1A1, DUOXA1, CYP2C42, CYB5A, CYP1A2, MAN1A1, GUCY2C, CDS1, RTN1, DTNBP1, TLR9, CYP4A21, INSIG2, FITM1, FMO1, FKBP14, SDR16C5, DIO1, SAR1B, CYP4A24, DPEP1

GO:0016324~apical plasma membrane	21	1.02E-05	KCNMA1, ATP1B1, PLD1, MYO7A, DUOX2, AQP5, DUOX1, SLC7A9, AQP6, ENPEP, AQP2, ATP6V1A, DRAM2, SLC2A2, SLC22A4, CLDN1, CA4, S100G, MUC13, DPP4, DPEP1
GO:0009897~external side of plasma membrane	17	0.001136053	CD3E, CD8B, FCER2, ANPEP, MFGE8, ENPEP, CCR7, CD86, ACE, IL12RB1, CD19, IFNG, MS4A1, CD79B, CD79A, BMPR1A, CD200R1
GO:0005765~lysosomal membrane	15	0.003801106	PLD1, STARD3NL, GNA11, MYO7A, TLR3, ANPEP, ENPEP, ATP6V1C1, LNPEP, ATP6V1A, NPC1, AP3M1, RNF152, CP, DPP4
GO:0031012~extracellular matrix	13	0.004535706	ZP2, ZP3, ADAMTS20, NDP, CILP, MFGE8, VTN, MMP3, MMP12, MMP1, APOE, F3, SLPI
GO:0072562~blood microparticle	12	3.41E-04	C8A, APOA4, APOA2, APOA1, APOE, ALB, APOA5, ITIH4, HRG, CD5L, CP, ANGPTL4
GO:0005923~bicellular tight junction	10	0.008453262	F11R, OCLN, BVES, CLDN5, LOC396566, CLDN1, CLDN22, CLDN10, CLDN20, CLDN14

Supplemental table 3. Celluar component in differentially expressed genes (LPS vs LPS+NAC)

Term	Count	PValue	Genes
GO:0005125~cytokine activity	37	8.46E-17	CSF2, NDP, GDF5, CD70, IFN-ALPHA-16, IFN-ALPHA-13, IFN-ALPHA-14, IFNA1, IFN-ALPHA-4, IFNG, IFN-ALPHA-5, IFN-OMEGA-6, IFN-OMEGA-4, IFN-DELTA-11, IFN-OMEGA-5, IFN-DELTA-10, IFN-OMEGA-2, LTB, IL1A, LTA, IL6, IFN-DELTA-9, IL7, IFN-DELTA-5, IFN-DELTA-7, INHA, LOC100153661, IFN-DELTA-3, LOC100155760, IFN-DELTA-4, INHBA, IL1B2, IL1B1, GDF11, IL12A, BMP7, BMP5
GO:0020037~heme binding	19	5.27E-05	CYP24A1, CYP1A1, PTGS2, CYP2C42, DUOX2, DUOX1, CYB5A, CYP2A19, CYP1A2, CYP2B22, CYP2J34, CYP4A21, CYP39A1, HRG, JAK2, CAT, NOS2, CYP4A24, CYP2C49
GO:0005132~type I interferon receptor binding	18	6.96E-12	IFN-DELTA-9, IFN-DELTA-5, IFN-DELTA-7, LOC100153661, IFN-ALPHA-16, IFN-DELTA-3, LOC100155760, IFN-DELTA-4, IFN-ALPHA-13, IFN-ALPHA-14, IFN-ALPHA-4, IFN-ALPHA-5, IFN-OMEGA-6, IFN-OMEGA-4, IFN-DELTA-11, IFN-OMEGA-5, IFN-DELTA-10, IFN-OMEGA-2
GO:0005506~iron ion binding	15	0.007477803	XDH, CYP24A1, CYP4A21, CYP2J34, CYP39A1, CYP1A1, CYP2C42, AOX1, AOX2, LTF, CYP2A19, CYP1A2, CYP4A24, CYP2B22, CYP2C49
GO:0005215~transporter activity	14	0.001102183	SLC5A4, CRABP1, RBP2, SAL1, SLCO2A1, LCN2, SLC11A1, FABP3, FABP1, FABP2, FABP7, SLC46A1, FABP6
GO:0016491~oxidoreductase activity	14	0.001726206	AKR1CL1, AKR1C4, AKR1E2, MAOA, F8, FADS3, FDXR, CYBRD1, GSTO1, LOC733635, SARDH, AKR1C1, CRYZL1
GO:0005179~hormone activity	12	1.73E-04	GCG, CGA, INHBA, EDN3, TTR, HAMP, FBN1, CRSP3, GHRL, ADM5, GAST, INHA
GO:0008289~lipid binding	12	2.48E-04	APOA4, PPARA, BPIFB1, STARD4, BPIFA1, FABP3, FABP1, PEX3, FABP7, PLTP, MTTP
GO:0004867~serine-type endopeptidase inhibitor activity	11	0.004469242	SERPINA7, WAP-1, E4, ITIH4, SERPINB10, SLPI, UFBP, SERPINA3-2, ITIH2, HRG, APLP2

Supplemental table 4. KEGG pathway in differentially expressed genes (LPS vs LPS+NAC)

Term	Count	PValue	Genes
ssc04060:Cytokine-cytokine receptor interaction	39	2.26E-08	IL1R2, CSF2, CCL2, BMPR2, IL-6, CD70, CCL5, CCL4, CCL27, IFN-ALPHA-8, IFN-ALPHA-13, CCL25, IFNA1, IFN-ALPHA-15, IFN-ALPHA-9, IL12RB1, CCL20, IFNG, LTB, IL1A, LTA, GHR, IL6, TNFSF4, IL7, CCL19, LOC100153661, CCR9, ACVR2A, TNFSF10, ACVR2B, CCR7, IL1B2, IL1B1, IL12A, EPOR, BMPR1B, BMP7, BMPR1A
ssc05168:Herpes simplex infection	33	1.79E-07	RNASEL, IFIH1, CCL2, TLR3, IL-6, OAS1, CCL5, TLR9, IFN-ALPHA-8, IFN-ALPHA-13, CASP3, IFNA1, IFN-ALPHA-15, IFN-ALPHA-9, PKR, IFNG, CASP8, TRAF6, CHUK, LTA, CDK1, IL6, SKP2, ARNTL, STAT1, LOC100153661, STAT2, IFIT1, IL1B2, IRF7, IL1B1, IL12A, JAK2
ssc05162:Measles	30	4.00E-09	IFIH1, IL-6, OAS1, TLR9, IFN-ALPHA-8, IRAK4, IFN-ALPHA-13, IFNA1, IFN-ALPHA-15, IFN-ALPHA-9, PKR, CD46, IFNG, TRAF6, IL1A, CHUK, AKT3, PIK3CG, IL6, CD3E, STAT1, LOC100153661, STAT2, TNFSF10, IL1B2, FCGR2B, IRF7, IL1B1, IL12A, JAK2
ssc05164:Influenza A	29	2.85E-06	RNASEL, IFIH1, CCL2, TLR3, IL-6, OAS1, CCL5, IFN-ALPHA-8, IRAK4, IFN-ALPHA-13, IFNA1, IFN-ALPHA-15, IFN-ALPHA-9, PKR, IFNG, CASP1, AKT3, IL1A, PIK3CG, IL6, STAT1, LOC100153661, STAT2, TNFSF10, IL1B2, IRF7, IL1B1, IL12A, JAK2
ssc04620:Toll-like receptor signaling pathway	27	6.99E-10	IL-6, PMAP-36, TLR9, IRAK4, IFN-ALPHA-8, IFN-ALPHA-13, CASP3, IFNA1, IFN-ALPHA-15, IFN-ALPHA-9, IFNG, CASP8, NOS2, TRAF6, IL1A, AKT3, IL6, CREB1, STAT1, LOC100153661, CASP10, FCGR2B, IL1B2, IL1B1, IL12A, JAK2, CD14

ssc05160:Hepatitis C	27	1.19E-07	PPARA, RNASEL, OCLN, CLDN5, LOC396566, TLR3, OAS1, CLDN10, CLDN14, IFN-ALPHA-8, IFN-ALPHA-13, IFNA1, IFN-ALPHA-15, IFN-ALPHA-9, PKR, TRAF6, CHUK, AKT3, PIK3CG, CLDN22, CLDN20, STAT1, LOC100153661, STAT2, IFIT1, IRF7, CLDN1
ssc05152:Tuberculosis	27	5.24E-05	TLR3, IL-6, TLR5, CCL5, CCL4, TLR8, TLR9, IRAK4, IFN-ALPHA-8, IFN-ALPHA-13, IFNA1, IFN-ALPHA-15, IFN-ALPHA-9, CASP8, TRAF6, CHUK, AKT3, PIK3CG, IL6, STAT1, LOC100153661, CD86, IL1B2, IRF7, IL1B1, IL12A, CD14
ssc03320:PPAR signaling pathway	26	1.52E-13	ACOX1, PPARA, ATP1B1, DRD2, GLI1, EDNRA, ATP2B1, HTR1B, BDNF, ADCY10, HTR1D, AKT3, PIK3CG, PLD1, CREB1, ATP1A2, TNNI3, SSTR2, NPY, CHRM2, CHRM1, PLN, GHRL, GHSR, LIPE, GLP1R
ssc04630:Jak-STAT signaling pathway	26	3.05E-06	CSF2, IL-6, STAT6, IFN-ALPHA-8, IFN-ALPHA-13, IFNA1, IFN-ALPHA-9, IFN-ALPHA-15, IL12RB1, IFNG, AKT3, GHR, IL13RA2, PIK3CG, IL6, IL7, SOCS7, SOCS4, SOCS5, STAT1, LOC100153661, STAT2, IL12A, JAK2, EPOR
ssc04024:cAMP signaling pathway	26	0.001019931	ACOX1, PPARA, AQP7, MMP1, APOA2, APOA1, PLIN1, APOC3, APOA5, CYP4A24, ACSL3, PLTP, ACSL5, ANGPTL4, ACADM, PCK2, CPT1A, PCK1, CD36, FABP3, FABP1, FABP2, GK, FABP7, SCP2, FABP6
ssc05161:Hepatitis B	25	3.67E-05	PIK3CG, IL6, IFIH1, EGR2, CREB1, TLR3, IL-6, RB1, LOC100153661, STAT1, STAT2, IFN-ALPHA-8, STAT6, CASP10, IFN-ALPHA-13, CASP3, IFNA1, IFN-ALPHA-9, IFN-ALPHA-15, IRF7, CASP8, CCNA2, AKT3, CHUK
ssc04062:Chemokine signaling pathway	22	0.004054441	AMCF-II, CCL1, PIK3CG, CCL2, CCL19, STAT1, CCL5, CCL4, CCL27, CCL17, STAT2, CCR9, CCL25, CCR7, CCL14, CCL20, RASGRP2, JAK2, GNB3, CRK, AKT3, CHUK
ssc04640:Hematopoietic cell lineage	20	5.65E-07	PIK3CG, IL6, CCL2, CD3E, GNA11, IL-6, CCL5, TLR9, IRAK4, ACE, IL1B2, GNAQ, CASP8, IFNG, IL1B1, IL12A, NOS2, TRAF6, AKT3, CHUK

ssc05142:Chagas disease (American trypanosomiasis)	20	5.10E-05	IL1R2, CSF2, IL6, IL7, CD3E, CD8B, FCER2, CD1.1, IL-6, ANPEP, GP9, CD36, CD19, IL1B2, CD59, IL1B1, MS4A1, EPOR, CD14, IL1A
ssc04668:TNF signaling pathway	19	1.86E-04	PIK3CG, CSF2, IL6, CCL2, PTGS2, CREB1, IL-6, MMP3, CCL5, CASP10, CASP3, IL1B2, CCL20, CASP8, IL1B1, SELE, LTA, AKT3, CHUK
ssc05323:Rheumatoid arthritis	18	3.06E-05	PIK3CG, IL1R2, CSF2, IL6, GNA11, IL-6, ACTN2, C8A, C8B, CASP3, IL1B2, GNAQ, IL1B1, IFNG, IL12A, SERPINB10, NOS2, CD14
ssc05146:Amoebiasis	18	3.55E-04	AMCF-II, CSF2, IL6, CCL2, IL-6, MMP3, CCL5, MMP1, ATP6V1C1, ATP6V1A, CD86, IL1B2, CCL20, IL1B1, IFNG, ANGPT1, LTB, IL1A
ssc04152:AMPK signaling pathway	17	0.003607132	PIK3CG, PRKAG3, CREB1, PRKAB2, PFKFB1, ADIPOR2, FBP2, PCK2, CPT1A, PCK1, CD36, GYS2, ADRA1A, PPP2R5E, CCNA2, LIPE, AKT3
ssc04920:Adipocytokine signaling pathway	16	3.96E-05	PRKAG3, PPARA, PRKAB2, ADIPOR2, ACACB, PCK2, CPT1A, PCK1, CD36, NPY, JAK2, AGRP, ACSL3, AKT3, CHUK, ACSL5
ssc00830:Retinol metabolism	15	5.90E-06	PRKAG3, LDHC, PPARA, CREB1, PFKFB1, PRKAB2, ACACB, PCK2, CPT1A, PCK1, GCG, GNAQ, SLC2A2, GYS2, AKT3
ssc04623:Cytosolic DNA-sensing pathway	15	2.68E-05	KCNMA1, TRPC1, ATP1B1, PNLIPRP1, CLCA1, ATP1A2, SCTR, ATP2B1, GNAQ, ATP2A1, PLA2G12B, CELA2A, SLC4A4, PLA2G2D, PLA2G2F
ssc04972:Pancreatic secretion	15	0.001204216	IL6, IL-6, LOC100153661, CCL5, CCL4, IFN-ALPHA-8, IFN-ALPHA-13, IFNA1, IFN-ALPHA-9, IFN-ALPHA-15, IL1B2, IRF7, IL1B1, CASP1, CHUK
ssc04922:Glucagon signaling pathway	15	0.003169166	RDH12, UGT1A6, LRAT, CYP1A1, CYP2C42, UGT2B31, AOX1, AOX2, SDR16C5, CYP2A19, CYP1A2, RDH16, CYP4A24, CYP2B22, CYP2C49
ssc05204:Chemical carcinogenesis	13	4.00E-04	AMCF-II, IL6, IL-6, IRAK4, CASP3, IL1B2, IL1B1, IL12A, NOS2, CASP1, TRAF6, IL1A, CD14

ssc05321:Inflammatory bowel disease (IBD)	13	4.70E-04	IFIH1, LOC100153661, IFN-ALPHA-8, IFN-ALPHA-13, CASP10, IFNA1, IFN-ALPHA-15, IFN-ALPHA-9, IRF7, CASP8, IL12A, TRAF6, CHUK
ssc00590:Arachidonic acid metabolism	13	5.49E-04	GPX2, PLA2G16, CYP2J34, PTGS2, CYP2C42, PLA2G12B, EPHX2, GGT1, PLA2G2D, CYP2B22, CYP4A24, CYP2C49, PLA2G2F
ssc04622:RIG-I-like receptor signaling pathway	13	0.001295649	STAT6, IL6, IL12RB1, IL1B2, IFNG, IL1B1, IL12A, IL-6, TLR5, FOXP3, STAT1, IL1A
ssc05133:Pertussis	13	0.002433038	GSTA1, GSTA2, CYP1A1, PTGS2, CYP2C42, CYP1A2, CYP2B22, UGT1A6, LOC780435, LOC100514231, UGT2B31, GSTO1, CYP2C49
ssc00982:Drug metabolism - cytochrome P450	12	2.41E-04	KCNMA1, ATP2B1, KCNN4, ATP1B1, GNAQ, LYZ, AQP5, ADRA1A, ATP1A2, PMAP-36, DMBT1
ssc04970:Salivary secretion	12	0.007941387	GSTA1, UGT1A6, GSTA2, LOC780435, LOC100514231, FMO1, MAOA, UGT2B31, AOX1, AOX2, CYP1A2, GSTO1
ssc05143:African trypanosomiasis	11	3.95E-05	IRAK4, IL1B2, PTGS2, IFNG, IL1B1, IL12A, JAK2, NOS2, TRAF6, STAT1, IL1A
ssc05140:Leishmaniasis	11	0.006272287	CASP3, IL6, IL1B2, IL1B1, CASP8, IL12A, IL-6, TLR5, SAR1B, CASP1, CD14
ssc05134:Legionellosis	11	0.006272287	IL6, APOA1, GNAQ, IL1B2, THOP1, IFNG, IL1B1, IL12A, IL-6, SELE, TLR9
ssc04975:Fat digestion and absorption	10	2.36E-04	IL6, CCL2, CD36, IL1B2, IFNG, IL1B1, IL12A, IL-6, SELE, TLR9
ssc04621:NOD-like receptor signaling pathway	10	0.004455668	IL6, CCL2, IL1B2, IL1B1, CASP8, IL-6, CCL5, TRAF6, CASP1, CHUK
ssc05144:Malaria	10	0.005829896	APOA4, PNLIPRP1, CD36, APOA1, PLA2G12B, FABP1, FABP2, PLA2G2D, PLA2G2F, MTTP

Supplemental table 5. Biological process in differentially expressed genes (Control vs LPS)

Term	Count	PValue	Genes
GO:0051607~defense response to virus	41	7.99E-26	CXCL9, IFN-ALPHA-17, IFN-ALPHA-16, CXCL10, TLR9, IFN-ALPHA-8, IFN-ALPHA-13, IFN-ALPHA-14, IFNA1, IFN-ALPHA-15, IFN-ALPHA-9, IFN-ALPHA-4, IFN-ALPHA-10, IFN-ALPHA-5, IFN-OMEGA-6, IFN-DELTA-11, IFN-OMEGA-4, LOC100153325, IFN-OMEGA-5, IFN-DELTA-10, IFN-ALPHA-1, LOC100156936, MX1, IFN-OMEGA-2, MX2, LOC100157354, IFN-DELTA-9, BST2, IFN-DELTA-5, IFN-DELTA-6, IFN-DELTA-7, IFN-DELTA-8, CD40, NLRP3, IFN-DELTA-1, LOC100153661, IFN-DELTA-2, IFN-DELTA-3, IFN-DELTA-4, OASL, IL12B
GO:0006955~immune response	29	3.43E-08	C7, CXCL2, CXCL9, IL-6, IL13, CXCL11, CCL27, IL10, CXCL10, SLA-1, CCL3L1, THBS1, LTB, CD28, IL6, LOC100622689, IL7, CCL19, GZMB, CD40, C8A, C8B, OASL, CCR7, CD36, IL1B2, IL1B1, CD274, IL12B
GO:0045087~innate immune response	28	3.68E-09	S100A8, S100A9, TLR2, IFN-ALPHA-16, TLR9, IFN-ALPHA-13, IFN-ALPHA-14, IFN-ALPHA-4, IFN-ALPHA-5, IFN-OMEGA-6, IFN-OMEGA-4, IFN-DELTA-11, IFN-DELTA-10, IFN-ALPHA-1, IFN-OMEGA-5, IFN-OMEGA-2, PTX3, MX2, IFN-DELTA-9, IFN-DELTA-5, FADD, IFN-DELTA-7, LOC100153661, IFN-DELTA-3, IFN-DELTA-4, S100A12, LOC692190, CD14
GO:0006954~inflammatory response	26	5.57E-07	CCL2, PTGS2, CRHBP, CXCL2, TLR2, CXCL9, IL13, CXCL11, IL10, TLR9, CXCL10, IL17A, CCL3L1, THBS1, PTGER3, HYAL3, CHI3L1, CCL19, CD40, NLRP3, S100A12, GPR33, CCR7, CCR3, BMPR1B, CD14

GO:0019221~cytokine-mediated signaling pathway	25	1.81E-11	CCL2, IFN-DELTA-9, IFN-DELTA-5, IFN-DELTA-7, DCN, LOC100153661, IFN-ALPHA-16, IFN-DELTA-3, IFN-DELTA-4, IL12RB2, IFN-ALPHA-13, IFN-ALPHA-14, IL1B2, IFN-ALPHA-4, IFN-ALPHA-5, IL1B1, IFN-OMEGA-6, IFN-DELTA-11, IFN-OMEGA-4, IFN-OMEGA-5, IFN-ALPHA-1, IFN-DELTA-10, IL12B, IFN-OMEGA-2, GHR
GO:0006959~humoral immune response	21	2.27E-14	TNF, IFN-DELTA-9, IL7, IFN-DELTA-5, IFN-DELTA-7, LOC100153661, IFN-ALPHA-16, IFN-DELTA-3, IFN-DELTA-4, IFN-ALPHA-13, IFN-ALPHA-14, IFN-ALPHA-4, IFN-ALPHA-5, IFN-OMEGA-6, IFN-OMEGA-4, IFN-DELTA-11, IFN-OMEGA-5, IFN-DELTA-10, IFN-ALPHA-1, IFN-OMEGA-2, LTA
GO:0042100~B cell proliferation	20	8.62E-15	IFN-DELTA-9, IFN-DELTA-5, LEF1, IFN-DELTA-7, IL7R, LOC100153661, IFN-ALPHA-16, IFN-DELTA-3, IFN-DELTA-4, IFN-ALPHA-13, IFN-ALPHA-14, IFN-ALPHA-4, IFN-ALPHA-5, IFN-OMEGA-6, IFN-OMEGA-4, IFN-DELTA-11, IFN-OMEGA-5, IFN-ALPHA-1, IFN-DELTA-10, IFN-OMEGA-2
GO:0030183~B cell differentiation	20	1.05E-11	IFN-DELTA-9, IFN-DELTA-5, IFN-DELTA-7, LOC100153661, IFN-ALPHA-16, IFN-DELTA-3, IFN-DELTA-4, DCLRE1C, IFN-ALPHA-13, IFN-ALPHA-14, IFN-ALPHA-4, IFN-ALPHA-5, IFN-OMEGA-6, CD79B, IFN-OMEGA-4, IFN-DELTA-11, IFN-OMEGA-5, IFN-ALPHA-1, IFN-DELTA-10, IFN-OMEGA-2
GO:0002323~natural killer cell activation involved in i	18	7.08E-14	IFN-DELTA-9, IFN-DELTA-5, IFN-DELTA-7, LOC100153661, IFN-ALPHA-16, IFN-DELTA-3, IFN-DELTA-4, IFN-ALPHA-13, IFN-ALPHA-14, IFN-ALPHA-4, IFN-ALPHA-5, IFN-OMEGA-6, IFN-OMEGA-4, IFN-DELTA-11, IFN-OMEGA-5, IFN-ALPHA-1, IFN-DELTA-10, IFN-OMEGA-2
GO:0002286~T cell activation involved in immune resp	18	1.20E-13	IFN-DELTA-9, IFN-DELTA-5, IFN-DELTA-7, LOC100153661, IFN-ALPHA-16, IFN-DELTA-3, IFN-DELTA-4, IFN-ALPHA-13, IFN-ALPHA-14, IFN-ALPHA-4, IFN-ALPHA-5, IFN-OMEGA-6, IFN-OMEGA-4, IFN-DELTA-11, IFN-OMEGA-5, IFN-ALPHA-1, IFN-DELTA-10, IFN-OMEGA-2

GO:0033141~positive regulation of peptidyl-serine pho	18	1.20E-13	IFN-DELTA-9, IFN-DELTA-5, IFN-DELTA-7, LOC100153661, IFN-ALPHA-16, IFN-DELTA-3, IFN-DELTA-4, IFN-ALPHA-13, IFN-ALPHA-14, IFN-ALPHA-4, IFN-ALPHA-5, IFN-OMEGA-6, IFN-OMEGA-4, IFN-DELTA-11, IFN-OMEGA-5, IFN-ALPHA-1, IFN-DELTA-10, IFN-OMEGA-2
GO:0043330~response to exogenous dsRNA	18	4.68E-12	IFN-DELTA-9, IFN-DELTA-5, IFN-DELTA-7, LOC100153661, IFN-ALPHA-16, IFN-DELTA-3, IFN-DELTA-4, IFN-ALPHA-13, IFN-ALPHA-14, IFN-ALPHA-4, IFN-ALPHA-5, IFN-OMEGA-6, IFN-OMEGA-4, IFN-DELTA-11, IFN-OMEGA-5, IFN-ALPHA-1, IFN-DELTA-10, IFN-OMEGA-2
GO:0002250~adaptive immune response	18	6.05E-11	IFN-DELTA-9, IFN-DELTA-5, IFN-DELTA-7, LOC100153661, IFN-ALPHA-16, IFN-DELTA-3, IFN-DELTA-4, IFN-ALPHA-13, IFN-ALPHA-14, IFN-ALPHA-4, IFN-ALPHA-5, IFN-OMEGA-6, IFN-OMEGA-4, IFN-DELTA-11, IFN-OMEGA-5, IFN-ALPHA-1, IFN-DELTA-10, IFN-OMEGA-2
GO:0042742~defense response to bacterium	14	2.51E-06	NPG1, LOC692190, NPG3, NPG4, PR39, FCGR1A, LYZ, ROMO1, PMAP-23, HP, PMAP-36, IL10, LEAP2, PG-2
GO:0055114~oxidation-reduction process	14	0.006825286	LDHC, CYB561, FTH1, SOD2, IYD, CYP4A21, DHRS4, DIO2, DIO3, MIOX, NOS3, CCS, DIO1, CYP4A24
GO:0007218~neuropeptide signaling pathway	12	4.08E-05	RXFP4, HCRTR1, SSTR2, PRLHR, CYSLTR2, NPW, GALP, PYY, POMC, AGRP, GPR1, ADCYAP1
GO:0007267~cell-cell signaling	12	2.33E-04	NTF3, FGF9, CCL3L1, IL7, ADRA1A, GJA1, ADRA2B, PYY, LHB, ADRA1D, SHH, IHH
GO:0006508~proteolysis	12	0.004139457	CLCA1, THOP1, ANPEP, LOC100302368, PAG6, CELA2A, ENPEP, CPB1, CAPN3, PPAG3, PLG, DPP4
GO:0001649~osteoblast differentiation	11	2.09E-04	BMP3, CYP24A1, BGLAP, FGF9, CCL3L1, RSPO2, LEF1, GJA1, VCAN, MYOC, SPP1
GO:0001525~angiogenesis	11	0.007735913	FGF9, TGFA, NOS3, ANPEP, ANGPT1, ADM2, MFGE8, ENPEP, ANGPT2, ENG, ANGPTL4

Supplemental table 6. Molecular function in differentially expressed genes (Control vs LPS)

Term	Count	PValue	Genes
GO:0005125~cytokine activity	39	8.38E-24	TNF, NDP, TGFB3, IL13, IFN-ALPHA-16, IL10, TIMP1, IFN-ALPHA-13, IFN-ALPHA-14, IFNA1, IL17A, IFN-ALPHA-4, IFN-ALPHA-5, IFN-OMEGA-6, IFN-DELTA-11, IFN-OMEGA-4, IFN-OMEGA-5, IFN-DELTA-10, IFN-ALPHA-1, IFN-OMEGA-2, LTB, LTA, SPP1, BMP3, IL6, IFN-DELTA-9, IL7, IFN-DELTA-5, IFN-DELTA-7, INHA, LOC100153661, IFN-DELTA-3, IFN-DELTA-4, INHBA, IL1B2, IL1B1, IL12B, BMP7, BMP5
GO:0005132~type I interferon receptor binding	18	2.58E-14	IFN-DELTA-9, IFN-DELTA-5, IFN-DELTA-7, LOC100153661, IFN-ALPHA-16, IFN-DELTA-3, IFN-DELTA-4, IFN-ALPHA-13, IFN-ALPHA-14, IFN-ALPHA-4, IFN-ALPHA-5, IFN-OMEGA-6, IFN-OMEGA-4, IFN-DELTA-11, IFN-OMEGA-5, IFN-ALPHA-1, IFN-DELTA-10, IFN-OMEGA-2
GO:0005506~iron ion binding	16	8.24E-05	XDH, CYP24A1, CYP1A1, CYP2A19, CYP1A2, CDO1, FTH1, CYP2J34, CYP4A21, P4HA1, AOX2, NOS3, CYP4A24, HBB, CYP2C49, ALOX12
GO:0005179~hormone activity	14	1.34E-07	FBN1, GAST, INHA, POMC, PTHLH, GCG, RETN, TTR, INHBA, ADM, GALP, GHRL, ADM5, LHB
GO:0020037~heme binding	13	0.001745905	CYP24A1, CYP1A1, PTGS2, STC2, CYP2A19, CYP1A2, CYP2J34, CYP4A21, HRG, NOS3, CYP4A24, HBB, CYP2C49
GO:0008083~growth factor activity	12	5.97E-06	INHBA, IL6, NTF3, FGF9, IL7, TGFB3, HBEGF, TGFA, IL12B, INHA, AREG, TIMP1
GO:0008201~heparin binding	11	0.00238143	REG4, FGF9, RSPO2, FBN1, HBEGF, HRG, CXCL11, THBS1, COL5A3, PLA2G2D, CXCL10

Supplemental table 7. Cellular component in differentially expressed genes (Control vs LPS)

Term	Count	PValue	Genes
GO:0005615~extracellular space	127	1.41E-43	S100A8, CRHBP, S100A9, EDN1, SNCA, TGFB3, IL13, LOC100302368, CXCL11, SHH, IL10, CXCL10, TTR, IFN-OMEGA-6, MS4A1, IFN-OMEGA-4, IFN-DELTA-11, IFN-DELTA-10, IFN-OMEGA-5, IFN-OMEGA-2, PPAG3, LOC100157354, CLCA1, STC2, MFGE8, LOC100153661, PTHLH, CBLN3, RETN, F5, STC1, XDH, BPIFB1, CCL2, CLU, GAST, IFN-ALPHA-17, POMC, IFN-ALPHA-16, TIMP1, IFN-ALPHA-13, IFNA1, IFN-ALPHA-14, IL17A, IFN-ALPHA-15, ALB, IFN-ALPHA-10, SERPINA3-2, AGRP, SPP1, PLAT, BMP3, EFEMP1, CHI3L1, IL1B2, IL1B1, AREG, PYY, BMP7, CPB1, LHB, BMP5, FGF9, UFBP, IL-6, ANPEP, IFN-ALPHA-8, NPG4, IFN-ALPHA-9, IFN-ALPHA-4, CCL3L1, IFN-ALPHA-5, TGFA, ANGPT1, LOC100153325, IFN-ALPHA-1, ANGPT2, LTB, LTA, IHH, IFN-DELTA-9, ZP3, CILP, IFN-DELTA-5, LYZ, IFN-DELTA-6, IFN-DELTA-7, INHA, IFN-DELTA-8, IFN-DELTA-1, IFN-DELTA-2, IFN-DELTA-3, IFN-DELTA-4, INHBA, ADM, CA6, GHRL, VCAN, IL12B, TG, TF, TNF, LUM, NDP, CXCL2, CXCL9, DCN, VCAM1, SERPINB10, ENO3, THBS1, PTX3, LOC100156936, MYOC, IL6, IL7, ICA, FBN1, CCL19, PAG6, GCG, AFP, ACE2, HBEGF, CELA2A, IGFBP4, IGFBP5

GO:0070062~extracellular exosome	86	0.008787243	RHOJ, LDHC, STEAP4, S100A8, SLC15A2, FGF9, S100A9, SAT2, CRABP2, AQP5, GJA1, PGAM2, ANPEP, ENPEP, CALB1, AQP2, ART3, TTR, ASPA, CXCR4, MIOX, MS4A1, ANGPT1, RPL11, ITIH3, TUBB1, PI16, DPP4, PEBP4, LYZ, CILP, MGP, MFGE8, FBP2, CD40, C8A, C8B, RETN, CTH, CA6, SCIN, RYR1, CA4, VMO1, PRNP, ZNHIT6, ALOX12, CAPS, TF, PRKCZ, C7, BPIFB1, FXYD3, BPIFB2, LUM, CLU, SFN, TIMP1, VCAM1, RPS28, PROCR, RPL7, ALB, UPK1A, ENO3, HRG, THBS1, AGRP, MYOC, MEST, SPP1, PLAT, GPD1, FBN1, EFEMP1, CHI3L1, KLK1, COL5A3, SLAMF1, CD19, IL1B1, CD274, CNFN, ACE2, CD79B, CP
GO:0005576~extracellular region	73	2.02E-26	WAP-3, MASP2, WAP-1, CRHBP, EDN1, FST, IL13, IL-6, HP, PMAP-36, CXCL10, NPG1, NPG3, NPG4, RSPO2, ANGPT1, ANGPT2, GHR, COL10A1, HYAL3, STC2, CRISP2, CA11, LYZ, PMAP-23, MFGE8, INHA, PG-2, RETN, INHBA, F5, GHRL, PLA2G2D, PLA2G2F, C7, PR39, PSP-I, GAST, TRH, POMC, LEAP2, CCL27, TIMP1, ADCYAP1, ZPBP, C1QTNF3, GALP, ACAN, AGRP, IL6, BGLAP, PNLIPRP2, LOC396757, NTF3, LOC396756, IL7, FBN1, CHI3L1, PLG, S100A12, SFRP5, LOC692190, SPAI-2, NPW, PON1, ADM5, ADM2, PYY, BMP7, LIPC, LHB, ASIP, BMP5
GO:0005887~integral component of plasma membrane	45	0.003179019	STEAP4, FXYD3, DRD1, PRLHR, TNF, CYSLTR2, SHAS2, AQP5, TSPAN9, CD72, GPR1, AQP2, ATP2B1, HCRTR1, HTR1B, UPK1A, MS4A1, TGFA, ADRA2B, SLC22A2, TRPC1, RXFP4, TRPC4, PTGER3, TRPC3, ATP4A, GPR75, SLC22A7, CD40, SLC7A14, EPHA3, NTRK3, GPR33, EPHA4, SLC26A5, SLC7A2, HAS1, HTR7, ATP2A1, PSEN2, CLDN1, HBEGF, ADRA1A, CD79B, ADRA1D
GO:0009897~external side of plasma membrane	24	7.01E-10	TNF, CXCL9, IL13, ANPEP, MFGE8, ITGB3, ENPEP, CD40, IL7R, SLAMF1, CXCL10, IL12RB2, VCAM1, CCR7, IL17A, IL12RB1, CD19, FCGR1A, CD274, MS4A1, CD79B, THBS1, ENG, CD200R1

GO:0016324~apical plasma membrane	14	0.001360016	PRKCZ, TF, MYO7A, AQP5, GJA1, ITGB3, ENPEP, AQP2, UPK1A, SLC2A2, PSEN2, CLDN1, CA4, DPP4
GO:0005578~proteinaceous extracellular matrix	14	0.006379526	ZP3, LUM, FBN1, CILP, DCN, COL5A3, UNC-6, SHH, TIMP1, ACAN, ADAMTS1, VCAN, COL10A1, ANGPTL4
GO:0031012~extracellular matrix	12	0.001256135	ZP3, NDP, EFEMP1, CLU, CILP, TGFB3, MGP, VCAN, MFGE8, DCN, THBS1, MYOC

Supplemental table 8. KEGG pathway in differentially expressed genes (Control vs LPS)

Term	Count	PValue	Genes
ssc04060:Cytokine-cytokine receptor interaction	38	6.21E-12	CCL2, TNF, TNFRSF12A, CXCL9, TGFB3, IL-6, IL13, CXCL11, IL7R, CCL27, IL10, CXCL10, IL12RB2, IFN-ALPHA-8, IFN-ALPHA-13, IL17A, IFNA1, IFN-ALPHA-15, IFN-ALPHA-9, IL12RB1, CXCR4, CCL3L1, LTB, LTA, GHR, AMHR2, IL6, IL7, CCL19, CD40, LOC100153661, CCR7, IL1B2, CCR3, IL1B1, IL12B, BMPR1B, BMP7
ssc04080:Neuroactive ligand-receptor interaction	28	1.17E-04	DRD1, PRLHR, ADORA3, CYSLTR2, ADCYAP1R1, LPAR3, LOC100302368, GHRHR, S1PR2, HCRTR1, HTR1B, CHRNA9, S1PR5, ADRA2B, GHR, RXFP4, PTGER3, GZMA, PLG, SSTR2, B2R, P2RY11, HTR7, ADRA1A, GHSR, LHB, ADRA1D
ssc04151:PI3K-Akt signaling pathway	26	0.009875204	FGF9, TLR2, IL-6, LPAR3, ITGB3, IL7R, IFN-ALPHA-8, IFN-ALPHA-13, IFNA1, IFN-ALPHA-15, IFN-ALPHA-9, GYS2, NOS3, ANGPT1, THBS1, ANGPT2, GHR, SPP1, IL6, HSP90AA1, IL7, COL5A3, LOC100153661, CD19, PPP2R5E, IKBKB
ssc04620:Toll-like receptor signaling pathway	25	2.08E-11	IL6, TNF, CXCL9, TLR2, IL-6, FADD, CD40, CXCL11, LOC100153661, CXCL10, TLR9, IFN-ALPHA-8, IFN-ALPHA-13, IFNA1, IFN-ALPHA-9, IFN-ALPHA-15, IL1B2, CD80, CCL3L1, IRF7, IL1B1, IL12B, IKBKB, CD14, SPP1
ssc05152:Tuberculosis	22	5.62E-05	IL6, TNF, CEBPB, TGFB3, TLR2, IL-6, FADD, LOC100153661, PMAP-36, IL10, TLR9, IFN-ALPHA-8, IFN-ALPHA-13, IFNA1, IFN-ALPHA-9, IFN-ALPHA-15, IL1B2, FCGR1A, IL1B1, IL12B, CD14
ssc05164:Influenza A	20	2.77E-04	IL6, TNF, CCL2, IL-6, LOC100302368, NLRP3, LOC100153661, PLG, CXCL10, IFN-ALPHA-8, IFN-ALPHA-13, IFNA1, IFN-ALPHA-9, IFN-ALPHA-15, IL1B2, IRF7, IL1B1, IL12B, IKBKB, MX1

ssc05168:Herpes simplex infection	20	8.18E-04	IL6, TNF, CCL2, TLR2, IL-6, FADD, LOC100153661, TLR9, IFN-ALPHA-8, SLA-1, IFN-ALPHA-13, IFNA1, IFN-ALPHA-9, IFN-ALPHA-15, IL1B2, IRF7, IL1B1, IL12B, IKBKB, LTA
ssc05162:Measles	19	3.81E-05	IL6, TLR2, IL13, IL-6, LOC100153661, SLAMF1, TLR9, IFN-ALPHA-8, IFN-ALPHA-13, IFNA1, IFN-ALPHA-9, IFN-ALPHA-15, IL1B2, IRF7, IL1B1, IL12B, MX1, CD28
ssc04630:Jak-STAT signaling pathway	17	7.91E-04	IL6, IL7, IL-6, IL13, LOC100153661, IL7R, IL10, IFN-ALPHA-8, IL12RB2, IFN-ALPHA-13, IFNA1, IL12RB1, IFN-ALPHA-9, IFN-ALPHA-15, IL12B, GHR, IL13RA2
ssc04668:TNF signaling pathway	16	1.37E-04	IL6, TNF, CCL2, CEBPB, PTGS2, CXCL2, EDN1, IL-6, FADD, CXCL10, VCAM1, IL1B2, IL1B1, IKBKB, LTA
ssc05144:Malaria	15	6.45E-08	IL6, CCL2, TNF, TLR2, TGFB3, IL-6, CD40, IL10, TLR9, VCAM1, CD36, IL1B2, IL1B1, THBS1, HBB
ssc04640:Hematopoietic cell lineage	15	1.67E-05	IL6, TNF, IL7, IL-6, ANPEP, ITGB3, IL7R, CD19, CD36, IL1B2, FCGR1A, IL1B1, MS4A1, PCD1B, CD14
ssc05323:Rheumatoid arthritis	15	3.89E-05	IL6, CCL2, TNF, TGFB3, TLR2, IL-6, IL17A, CD80, IL1B2, CCL3L1, IL1B1, ANGPT1, LTB, CD28
ssc05146:Amoebiasis	15	3.18E-04	IL6, TNF, TGFB3, TLR2, IL-6, COL5A3, IL10, C8A, C8B, ARG1, IL1B2, IL1B1, SERPINB10, IL12B, CD14
ssc05142:Chagas disease (American trypanosomiasis)	15	4.28E-04	IL6, CCL2, TNF, TGFB3, TLR2, IL-6, FADD, IL10, TLR9, B2R, IL1B2, CCL3L1, IL1B1, IL12B, IKBKB
ssc05160:Hepatitis C	15	0.002341748	PPARA, TNF, CLDN5, LOC396566, CLDN22, LOC100153661, CLDN14, IFN-ALPHA-8, IFN-ALPHA-13, IFNA1, IFN-ALPHA-9, IFN-ALPHA-15, IRF7, CLDN1, IKBKB
ssc05320:Autoimmune thyroid disease	14	3.01E-07	IFN-ALPHA-8, TG, SLA-1, IFN-ALPHA-13, IFNA1, IFN-ALPHA-9, IFN-ALPHA-15, CD80, GZMB, CD40, LOC100153661, IL10, CD28
ssc04623:Cytosolic DNA-sensing pathway	14	3.49E-06	IL6, IL-6, LOC100153661, CXCL10, IFN-ALPHA-8, IFN-ALPHA-13, IFNA1, IFN-ALPHA-9, IFN-ALPHA-15, IL1B2, IRF7, IL1B1, IKBKB, ZBP1
ssc05321:Inflammatory bowel disease (IBD)	13	1.98E-05	IL12RB2, IL17A, IL6, IL12RB1, TNF, IL1B2, IL1B1, TGFB3, TLR2, IL13, IL-6, IL12B, IL10

ssc04622:RIG-I-like receptor signaling pathway	13	6.16E-05	IFN-ALPHA-8, IFN-ALPHA-13, IFNA1, IFN-ALPHA-9, TNF, IFN-ALPHA-15, IRF7, FADD, IL12B, IKBKB, LOC100153661, DHX58, CXCL10
ssc04350:TGF-beta signaling pathway	12	8.33E-04	AMHR2, INHBA, TNF, CDKN2B, FST, TGFB3, DCN, BAMBI, BMPR1B, BMP7, THBS1, BMP5
ssc05143:African trypanosomiasis	11	2.11E-06	VCAM1, IL6, TNF, IL1B2, THOP1, IL1B1, IL-6, IL12B, HBB, IL10, TLR9
ssc04064:NF-kappa B signaling pathway	11	0.008343059	VCAM1, TNF, IL1B2, PTGS2, IL1B1, CCL19, CD40, IKBKB, LTB, CD14, LTA
ssc05332:Graft-versus-host disease	10	1.79E-05	SLA-1, IL6, TNF, IL1B2, CD80, IL1B1, IL-6, GZMB, CD28
ssc04940:Type I diabetes mellitus	10	8.95E-05	SLA-1, TNF, IL1B2, CD80, IL1B1, GZMB, IL12B, LTA, CD28
ssc00830:Retinol metabolism	10	7.94E-04	RDH12, DHRS4, CYP1A1, AOX2, SDR16C5, CYP2A19, CYP1A2, PNPLA4, CYP4A24, CYP2C49

Supplemental table 9. list of primers

Gene symbol	Description	Acession No.	Primers (5' --> 3')	
			Forward	Reverse
HMGCS3	3-hydroxy-3-methylglutaryl-CoA synthase 2	NM_214380	CAGGACTCAGGCAACACTGA	GACAGCGATGTCTCCACAGA
LDHC	lactate dehydrogenase C	NM_001195775	ATTGTTTCAAATCCGGTGGA	TTCAGAGCAACACCAGCAAC
PHEROC	pheromaxein C subunit	NM_001123161	GCAGGTTCTGGCTGTTCCTA	CACTTGCATAAACACGCTGAA
TNNC2	troponin C2, fast skeletal type	NM_001001862	CCTACCTCAGCGAGGAGATG	TCTTCGAAGTCGATGGTTCC
LGALS13	lectin, galactoside-binding, soluble, 13	NM_001142841	GTCCCCTACACGAAGCATGT	AAGCTCACTCGGAAGTGGAA
XDHs	xanthine dehydrogenase	NM_001285974	AACAGCCTGGACCTCTCTCA	TCACTGCGACCTCACTCATC
MYH2	myosin, heavy chain 2, skeletal muscle, adult	NM_214136	GGAAAGTCGCAGAACAGGAG	CGAGCTTCCTGGAGAATGTC
SAL1	salivary lipocalin	NM_213814	ATGTCAATGGCGACAAAACA	AGTTGGAAGCAGCGATCAAT
SLC2A2	solute carrier family 2 member 2	NM_001097417	GGGTTCCTTCCAGTTTGGAT	GGCTAGCAGATGCCGTAGTC
SDR16C5	short chain dehydrogenase/reductase family 16C, member 5	NM_001195368	GGCAGAAAGTTCCTCGACTG	GCTGCAAATTTACTCGCACA
NF-κB	Nuclear factor of kappa light polypeptide gene enhancer in B-cells 1	NM_001048232	GACAACATCTCCTTGGCGGG	TCTGCTCCTGCTGCTTTGAGG
TNF-α	Tumor necrosis factor alpha	NM_214022	TCTCCTTCCTCCTGGTCGCA	TCCCTCGGCTTTGACATTGG
IFN-γ	Interferon gamma	NM_213948	GGCCATTCAAAGGAGCATGG	GATGGCTTTGCGCTGGATCT
IL-6	Interleukin-6	NM_214399	AGCCCACCAGGAACGAAAGA	AGCCATCACCAGAAGCAGCC
SOD1	Superoxide dismutase 1	NM_001190422	GTACCAGTGCAGGTCCTCAC	TTTGCCAGCAGTCACATTGC
GCLM	Glutamate-cysteine ligase modifier subunit	XM_001926378	TTGGAGCAGCTGTACCAGTG	GAGCTTCCTGGAAACTCGCT
GCLC	Glutamate-cysteine ligase catalytic subunit	XM_003482164	GTCCAGTTGGTCCTGTCTGG	CGGGAGTCCCTTCGATCATG
CAT	Catalase	NM_214301	ACACAGGCACATGAACGGAT	GTCCCGGATGCCATAGTCAG
ZO-1	Zonula occludens 1	XM_021098856	GATCCTGACCCGGTGTCTGA	TTGGTGGGTTTGGTGGGTTG
OCLN	Occludin	NM_001163647	GAGAGAGTGGACAGCCCCAT	TGCTGCTGTAATGAGGCTGC
MMP2	matrix metallopeptidase 2	NM_214192	ACTCCCACTTTGACGACGAT	CGTACTTGCCATCCTTGTCG
MMP9	matrix metallopeptidase 9	NM_001038004	TGAAGACGCAGAAGGTGGAT	TTCAGGAGGTCGAAGGTCAC
CDH1	cadherin 1	NM_001039258	TGTACGAAGGTGTGGTGGAA	AGCGGCTCTTGGTCTCATAA
RND3	Rho family GTPase 3	XM_422158	CAACATGTGGGACACCTCAG	ATTTGCAACCAACCAAAAGC
GAPDH	glyceraldehyde-3-phosphate dehydrogenase	NM_204305	GGTGGTGCTAAGCGTGTTAT	ACCTCTGTCATCTCTCCACA
